# Unique Role of Proximal Tubule Dipeptidyl Peptidase 4 on Blood Pressure, Renal Sodium Handling, and Na^+^/H^+^ Exchanger Isoform 3 Phosphorylation

**DOI:** 10.1111/apha.70127

**Published:** 2025-11-04

**Authors:** Flavia L. Martins, Joao Carlos Ribeiro‐Silva, Erika Fernandes de Jesus, Ravi Nistala, Adriana C. C. Girardi

**Affiliations:** ^1^ Departamento de Cardiopneumologia, Faculdade de Medicina Universidade de São Paulo São Paulo Brazil; ^2^ Division of Nephrology, Department of Medicine University of Missouri School of Medicine Columbia Missouri USA; ^3^ State University of New York (SUNY) Upstate Medical University Syracuse New York USA

**Keywords:** angiotensin II, blood pressure, dipeptidyl peptidase 4, natriuresis, proximal tubule, pS552‐NHE3

## Abstract

**Background:**

Dipeptidyl peptidase 4 (DPP4) is a transmembrane serine exopeptidase highly expressed in the proximal tubule (PT). While its enzymatic role is well characterized, its non‐enzymatic functions remain unclear. DPP4 physically associates with the Na^+^/H^+^ exchanger isoform 3 (NHE3), and DPP4 inhibitors promote natriuresis; however, the mechanisms by which DPP4 regulates NHE3 and its role in blood pressure (BP) regulation remain controversial. We hypothesized that PT DPP4 promotes sodium reabsorption and attenuates pressure–natriuresis by preventing NHE3 phosphorylation at serine 552 (pS552).

**Methods:**

We generated PT‐specific *Dpp4* knockout mice (*Dpp4* ^ΔPT^) and examined the effects of PT‐specific and global *Dpp4* deletion (*Dpp4* ^−/−^) on systolic blood pressure (SBP), natriuresis, and NHE3 phosphorylation at baseline and following acute angiotensin II (Ang II) infusion in male and female mice.

**Results:**

Both *Dpp4* ^ΔPT^ and *Dpp4* ^−/−^ showed enhanced diuretic and natriuretic responses to saline loading, with higher renal pS552‐NHE3, and unchanged baseline SBP. Ang II elevated DPP4 activity in controls but not in *Dpp4* ^ΔPT^ mice, suggesting that PT DPP4, rather than DPP4 in other nephron segments, is regulated by Ang II under these experimental conditions. Ang II increased SBP in all groups, but the pressor response was significantly attenuated in both *Dpp4* ^ΔPT^ and *Dpp4* ^−/−^ mice, paralleling sustained pS552‐NHE3 elevation.

**Conclusion:**

These findings demonstrate that DPP4 modulates NHE3 activity by preventing pS552‐NHE3 accumulation, promoting an anti‐natriuretic effect. In the absence of PT DPP4, these mechanisms are disrupted, reducing Ang II sensitivity, maintaining high pS552‐NHE3 levels, and likely enhancing pressure–natriuresis, underscoring the role of PT DPP4 in modulating signaling mechanisms governing renal function.

**Abbreviations**
Ang II: angiotensin II
AT1R: angiotensin II type 1 receptor
BP: blood pressure
DPP4: dipeptidyl peptidase 4
DPP4is: dipeptidyl peptidase 4 inhibitors
GLP‐1: glucagon‐like peptide‐1
NHE3: Na^+^/H^+^ exchanger isoform 3
PT: proximal tubule
RAS: renin‐angiotensin system
SHR: spontaneously hypertensive rat

## 1 Introduction

Dipeptidyl peptidase 4 (DPP4/CD26) is a widely expressed serine protease found in epithelial and non‐epithelial cells across various tissues, with particularly high levels in the kidney [1]. In renal tissue, DPP4 is localized in the glomeruli and the proximal tubule (PT), where it is a major component of the microvilli brush border [2, 3, 4]. In addition to its enzymatic activity, DPP4 is involved in a variety of biochemical pathways and physically associates with multiple proteins, including adenosine deaminase [5], caveolin [6], components of the extracellular matrix [7, 8], and the sodium‐hydrogen exchanger 3 (NHE3) [3, 9].

In the PT, NHE3 mediates approximately 70% filtered sodium reabsorption, playing a crucial role in extracellular volume homeostasis and blood pressure (BP) control [10, 11, 12]. Mice with PT‐specific deletion of *Slc9a3* (which encodes NHE3) display lower BP, enhanced pressure‐natriuresis, and attenuated hypertensive responses to chronic angiotensin II (Ang II) infusion compared to wild‐type controls [12, 13]. Notably, studies have shown that following the onset of hypertension, PT NHE3‐mediated sodium reabsorption declines [14, 15, 16], thereby limiting further BP increases [17, 18]. This reduction in NHE3 activity is thought to result from increased phosphorylation at serine 552, along with a redistribution of NHE3 from the body to the base of the PT microvilli [14, 19].

Previous work demonstrates that DPP4 inhibitors (DPP4is) downregulate PT NHE3 activity, leading to natriuresis [20, 21, 22]. However, the molecular mechanisms underlying this inhibition are not completely elucidated, and despite their natriuretic properties, the impact of DPP4is on BP remains inconclusive. While some studies reported BP reductions in individuals with mild hypertension [23], chronic kidney disease models [24], and pre‐hypertensive spontaneously hypertensive rats (SHRs) [25], findings in adult hypertensive animals have been mixed, with outcomes ranging from BP reduction to no change or even BP increases [25, 26, 27].

Given the limited understanding of the physiological role of PT DPP4 and the variable BP responses to DPP4is across different contexts, we generated mice with PT‐specific deletion of *Dpp4* and assessed BP, the response to acute saline loading, and renal NHE3 phosphorylation at serine 552 under both baseline conditions and during acute Ang II‐induced BP elevation. We hypothesized that PT DPP4 modulates BP by preventing the accumulation of pS552‐NHE3, thereby promoting sodium reabsorption and attenuating pressure–natriuresis. Because adaptive mechanisms may compensate for the absence of DPP4 under basal conditions, resulting in unchanged baseline BP, we used acute Ang II infusion, a potent vasoconstrictor, as a physiological challenge to evaluate whether PT DPP4 is required for a full pressor response. To distinguish the specific contribution of PT DPP4 from that of systemic DPP4, we conducted parallel experiments in global *Dpp4*‐deficient mice and examined potential sex differences in these regulatory mechanisms.

### 1.1 Experimental Animals

#### 1.1.1 Animals

All animal procedures were approved by the Institutional Animal Care and Use Committees at the University of Missouri and the University of São Paulo Medical School in accordance with the National Institutes of Health Guide for the Care and Use of Laboratory Animals. Experiments were conducted on 12‐week‐old male and female mice maintained on a 12:12 light/dark cycle under standard environmental conditions with *ad libitum* access to autoclaved water and standard chow. Heterozygous mice for global *Dpp4* deletion (*Dpp4* ^*+/−*^) were obtained from the Infrafrontier/European Mouse Mutant Archive (https://www.infrafrontier.eu/emma/) [28]. *Dpp4* ^*−/−*^ (homozygous knockout) and *Dpp4* ^*+/+*^ (wild type, WT) mice were obtained by crossing male and female *Dpp4* ^*+/−*^ mice. *Dpp4* heterozygous floxed mice (*Dpp4* ^*Fl/+*^), on a C57BL/6NTac background, were obtained from Taconic Biosciences (Model n. 10053, Rensselaer, NY) [29] and bred to generate homozygous litters (*Dpp4* ^*Fl/Fl*^ *). Dpp4* ^*Fl/Fl*^ mice were then crossed with iL‐*Sglt2‐Cre* mice [30] (obtained from the Jia L Zhuo lab, University of Mississippi Medical Center, Jackson, MS) to obtain homozygous PT‐*Dpp4* knockout mice and their Cre‐negative littermates. The experimental design and a CONSORT‐like diagram of the study are presented in Figures S1 and S2, respectively.

#### 1.1.2 Genotyping

Procedures followed Taconic Biosciences guidelines. Briefly, tail snips were digested for Direct PCR by incubation in tail lysis buffer (catalog #102‐T, Viagen Biotech) with proteinase K (catalog #503‐PK, Viagen Biotech) overnight at 65°C, followed by 1 h at 85°C. The lysates were centrifuged at 500 × g for 5 min at 4°C to obtain DNA. For PCR, the reaction mixture included 50% DreamTaq Green PCR Master Mix 2× (catalog #K1081, Thermo Fisher Scientific), 25 pmol each of forward and reverse primers, and 20 ng/μl DNA. The PCR was performed using a C1000 Touch Thermal Cycler (BIO‐RAD); conditions and primer sets (from Integrated DNA Technologies Inc.) are detailed in Table S1. PCR products were resolved on a 1% agarose gel in TAE buffer (40 mM Tris acetate, 10 mM EDTA, pH 8.0), stained with SYBR Safe (catalog #S33102, Invitrogen), and visualized against a 100 bp DNA ladder (GenDEPOT). Electrophoresis was run at 120 V for 25 min, and band images were digitized using Licor's Odyssey XF Imager and quantified with ImageStudio software (Figure S3).

#### 1.1.3 Systolic Blood Pressure (SBP) Determination

SBP was determined with a Hatteras Instruments MC4000 plethysmograph system (Grantsboro, NC), as previously published [31]. SBP was measured before and 40 min after Ang II administration (Figure S1). Briefly, mice were acclimated to restraint and tail‐cuff inflation for 10 min over six consecutive days. During the experiment, the restrainers' platform was set to heat at 38°C, ensuring sufficient blood flow for acquisition by the equipment, and the first five preliminary measurements were discarded. Eight to ten consecutive stable readings were used to determine mean SBP at baseline and following Ang II administration (Tables S2–S5). ΔSBP was determined by subtracting baseline SBP from post‐Ang II SBP measurements. All measurements were performed by an observer blind to the mice's genotype and treatment conditions.

#### 1.1.4 Acute Ang II Administration

To induce an acute increase in BP, Ang II (catalog #9525, Sigma, St. Louis, MO) was administered intraperitoneally, as reported in the literature [32], at a pressor dose of 1000 ng/kg/min (equivalent to 60 μg/kg) [33]. Kidneys were collected 1 h post‐injection for analysis, following full protocol validation in our laboratory (Figure S4). Saline was used as the control. All injections were performed by an observer blind to the mice's genotype and treatment condition. Mice were sedated with isoflurane (3%–4% for induction and 2.5%–3% for maintenance). The left kidney was immediately excised and placed in ice‐cold PBS buffer (150 mM sodium chloride, 2.8 mM monobasic sodium phosphate, 7.2 mM dibasic sodium phosphate, pH 7.4) containing protease inhibitors (0.7 μg/mL pepstatin, 0.5 μg/mL leupeptin and 40 μg/mL phenylmethanesulfonylfluoride) and phosphatase inhibitors (50 mM sodium fluoride and 15 mM sodium pyrophosphate) for subsequent homogenization. The right kidney was fixed in 4% paraformaldehyde in PBS (catalog # J61899, Thermo Fisher Scientific).

#### 1.1.5 Saline Challenge

Mice were anesthetized with isoflurane (3%–4% for induction, 2.5%–3% for maintenance) and received an intraperitoneal injection of 0.9% NaCl (37°C) at 10% of body weight (v/w) [34, 35, 36]. Mice's weight and absolute values of injected saline solution are described in Tables S6 and S7. They were then placed in Tecniplast metabolic cages (Buguggiate, Italy) and urine was collected for 5 h. Urinary sodium was measured with a Beckman Coulter AU480 analyzer, and excretion was expressed as a percentage of the injected sodium and fluid load.

#### 1.1.6 Immunofluorescence Analysis

The right kidney was fixed in 4% paraformaldehyde in PBS (catalog #J61899, Thermo Fisher Scientific) for 24 h, stored in 70% ethanol, and sent to a tissue processing facility for downstream processing and paraffin embedding. Tissue was sectioned at 4 μm and mounted on silanized slides (StarFrost, Knittel Glass, Bielefeld, Germany). Sections were deparaffinized, rehydrated, and underwent antigen retrieval by heating at 140°C for 20 min in 10 mM citrate buffer (pH 6.0) with 1% SDS. After two deionized water washes and demarcation with a hydrophobic barrier pen (PAP pen, catalog #AB2601, Abcam), nonspecific binding was blocked with 5% BSA and 0.25% Triton X‐100 in PBS for 30 min. Sections were incubated overnight at 4°C with primary antibodies against DPP4 (1:100, catalog #AF954, R&D Systems) and SGLT2 (1:100, catalog #20802, Bicell) in blocking solution. Following three 5‐min PBS washes, sections were incubated for 1 h at room temperature with Alexa Fluor 488‐conjugated donkey anti‐goat IgG (catalog #A11055, Life Technologies/Thermo Fisher Scientific), Alexa Fluor 647‐conjugated donkey anti‐rabbit IgG (catalog #A31573, Life Technologies/Thermo Fisher Scientific), and DAPI (catalog #62248, Thermo Fisher Scientific) diluted 1:500 in blocking solution. After three additional 5‐min PBS washes, sections were mounted in Fluoromount G (catalog #004958 02, Invitrogen/Thermo Fisher Scientific), coverslipped (Knittel Glass), and allowed to dry at room temperature before visualization with an EVOS M7000 microscope (Thermo Fisher Scientific). Identical fluorescent excitation and detection parameters were used across all experimental groups.

#### 1.1.7 Kidney Homogenate Preparation

The left kidney was homogenized in ice‐cold PBS containing protease and phosphatase inhibitors using a Potter–Elvehjem‐style tissue grinder (POLIMIX PX‐SR50E, Kinematica Inc., Luzern, Switzerland). The homogenate was then cleared by centrifugation (2400 × g for 10 min at 4°C), aliquoted, and stored at −80°C. Protein concentration was determined by the bicinchoninic acid (BCA) method using the Pierce BCA Protein Assay Kit (Thermo Fisher Scientific).

#### 1.1.8 SDS‐PAGE and Immunoblotting

Mouse kidney homogenates were solubilized in Laemmli sample buffer (catalog #1610737, Bio‐Rad) and separated using either commercial (Criterion TGX, catalog #5671125, Bio‐Rad) or homemade 10% SDS‐PAGE gels. Proteins were then transferred overnight at 4°C onto polyvinylidene fluoride membranes (catalog #88518, Thermo Fisher Scientific) using the Criterion Blotter with Wire Electrodes (catalog #17704071, Bio‐Rad) at 30 V for 16 h at 4°C. Membranes were blocked for 1 h in PBS containing 5% nonfat dry milk and 0.1% Tween‐20, then incubated overnight at 4°C with primary antibodies (see Table S8 for details). The following day, membranes were washed, incubated for 1 h at room temperature with the corresponding horseradish peroxidase–conjugated secondary antibodies, and washed again. Proteins were visualized using an enhanced chemiluminescence detection system (catalog #34095, SuperSignal West Femto Maximum Sensitivity Substrate, Thermo Fisher Scientific) on an Odyssey XF photodocumenter (Li‐Cor Biotechnology, Lincoln, NE) with ImageStudio software (Li‐Cor Biotechnology). Band intensities were quantified using Scion Image software (Scion, Frederick, MD), and results were normalized to Ponceau and expressed as a percentage of the control for each gel.

#### 1.1.9 DPP4 Activity Assay

Kidney DPP4 activity was evaluated in 100 μg of renal homogenates diluted in DPP4 assay buffer (Tris–HCl, 150 mM NaCl, pH 8.0). 50 μL of the diluted sample was added to a 96‐well black flat‐bottom plate. Next, 50 μL of the fluorescent DPP4 substrate, 100 μM H‐Ala‐Pro‐AFC (I‐680, Bachem, Torrence, CA), was added to each well and incubated for 10 min at room temperature, protected from light. Fluorescence was measured using the Synergy Microplate Reader (Biotek, Winooski, VT) with an excitation wavelength of 405 nm and an emission wavelength of 535 nm. The assays were conducted in duplicates in the presence and absence of the DPP4 inhibitor (10 μM linagliptin, Sigma). The result was calculated as relative light units (RLU) and presented as a percentage of the control group.

#### 1.1.10 Renal Ang II Content

Renal angiotensin II content was measured by Enzyme‐Linked ImmunoSorbent Assay (Biomatik, Cambridge, ON—catalog #EKU02406) as previously published [31, 37, 38] following the manufacturer's instructions.

#### 1.1.11 Statistical Analysis

**FIGURE 1:**
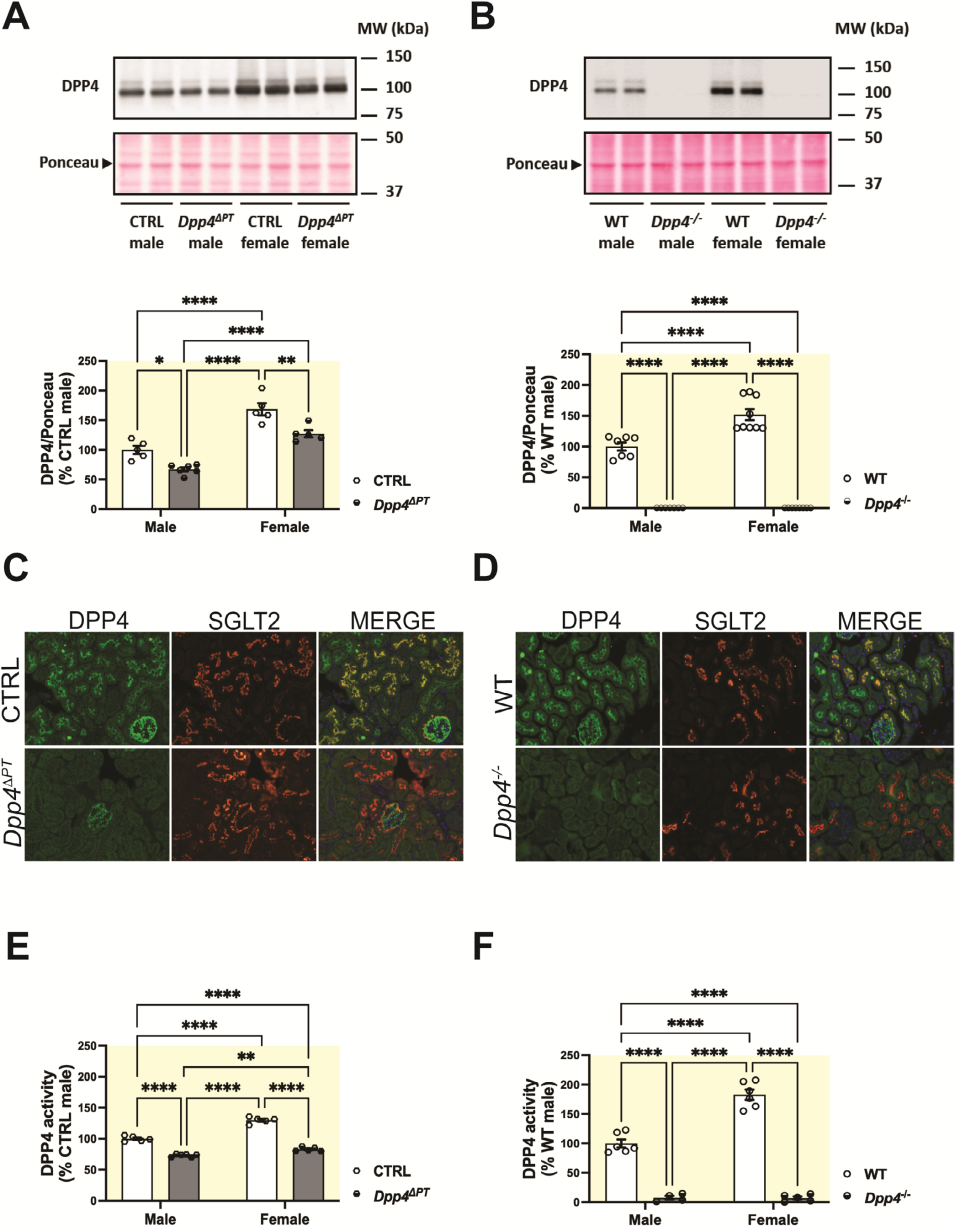
Phenotypic characterization of *Dpp4* ^ΔPT^ and *Dpp4* ^−/−^ mice. DPP4 protein abundance was evaluated by immunoblotting using equivalent amounts of 10 μg of renal homogenate samples from mice with either (A) PT‐specific (*Dpp4* ^ΔPT^) or (B) global *Dpp4* deletion (*Dpp4* ^−/−^) and their respective controls. Data normalized to Ponceau staining. Each dot represents the % of DPP4 expression relative to male CTRL or WT per animal. Representative images of the immunostaining of kidney sections for SGLT2, a PT marker, and DPP4 in (C) *Dpp4* ^ΔPT^ and (D) *Dpp4* ^−/−^ mice. Renal DPP4 activity was assessed by fluorimetry in renal homogenates from (E) *Dpp4* ^ΔPT^ and (F) *Dpp4* ^−/−^ mice. Each dot represents the % of DPP4 activity relative to male CTRL or WT per animal. Bars represent mean ± SEM. Data normality was assessed with the Shapiro–Wilk test. The experimental n ranged from 5 to 9. Statistical analysis was performed using two‐way ANOVA followed by Tukey's post‐test. *p < 0.05, **p < 0.01 and ****p < 0.0001.

**FIGURE 2:**
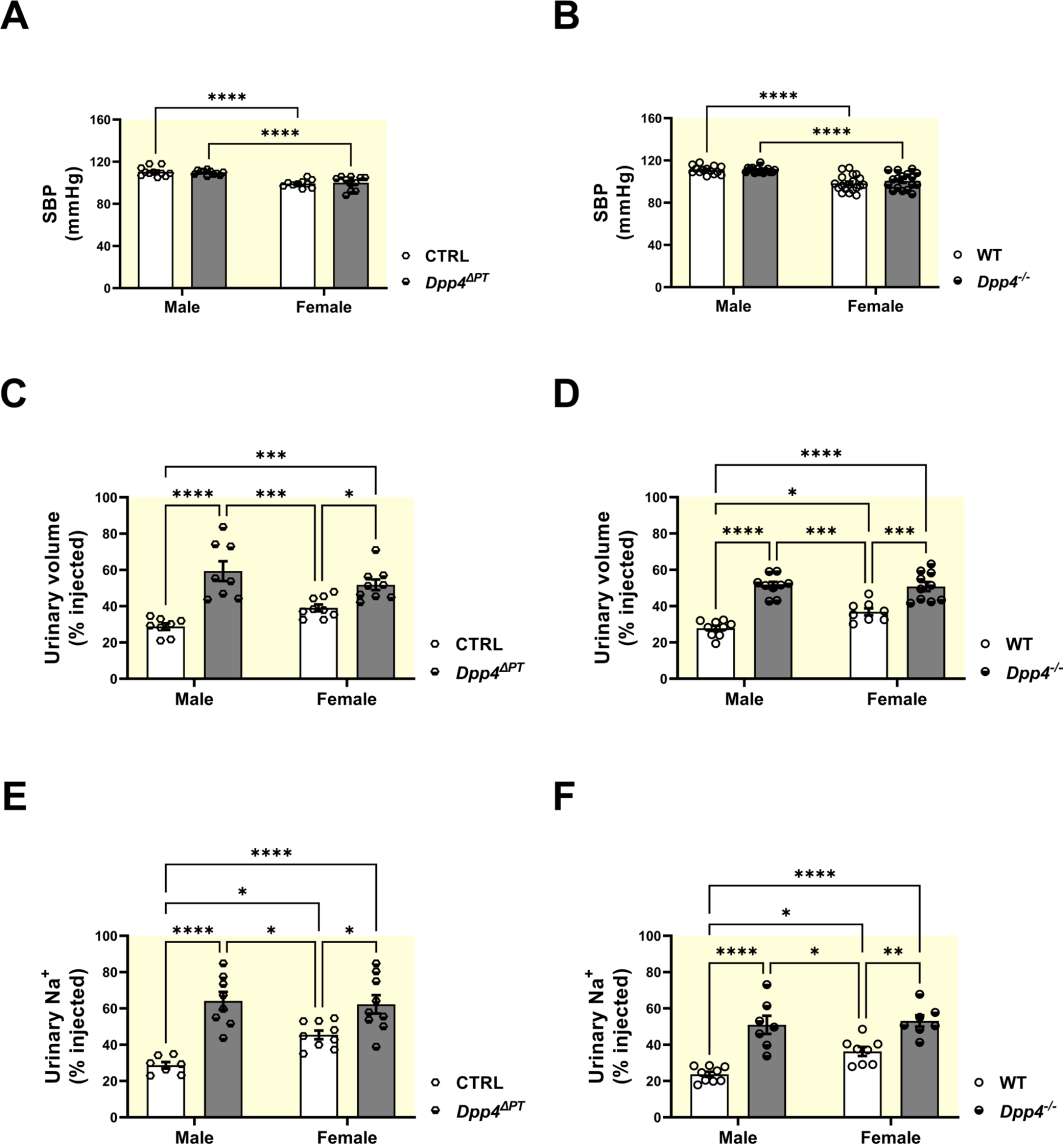
Blood pressure and acute natriuretic and diuretic responses in male and female *Dpp4* ^ΔPT^ and *Dpp4* ^−/−^ mice. Systolic blood pressure (SBP) was measured by tail‐cuff plethysmography in male and female (A) *Dpp4* ^ΔPT^ and (B) *Dpp4* ^−/−^ mice. Acute renal natriuretic and diuretic responses were evaluated after a saline challenge. Results expressed as (C, D) % of fluid load and (E, F) % sodium load excreted within 5 h. Each dot represents individual measurements. Data normality was assessed with the Shapiro–Wilk test. The experimental n ranged from 5 to 9 (SBP) or 7–10 (saline challenge). Statistical analysis was performed using two‐way ANOVA followed by Tukey's post‐test. Bars represent mean ± SEM. *p < 0.05, **p < 0.01, ***p < 0.001 and ****p < 0.0001.

**FIGURE 3:**
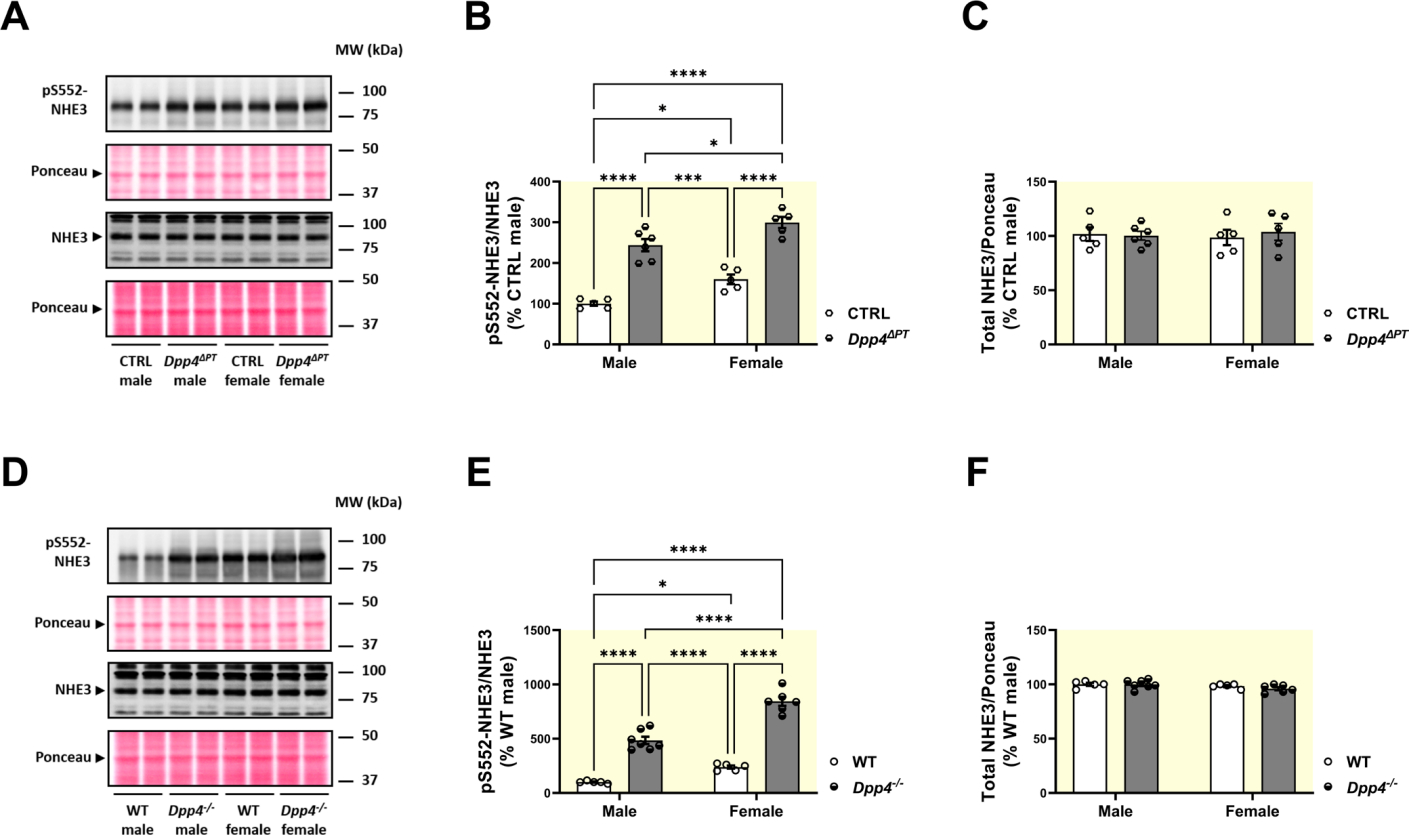
Effect of *Dpp4* deletion on kidney NHE3 phosphorylation in male and female *Dpp4* ^ΔPT^ and *Dpp4* ^−/−^ mice. Levels of phosphorylated (pS552‐NHE3) and total NHE3 were determined by immunoblotting in kidney homogenates from (A–C) *Dpp4* ^ΔPT^ and (D–F) *Dpp4* ^−/−^ mice. Data normalized to Ponceau staining. Each dot represents the % of pS552‐NHE3/NHE3 relative to male CTRL or WT per animal. Bars represent mean ± SEM. Data normality was assessed with the Shapiro–Wilk test. The experimental *n* ranged from 5 to 9. Statistical analysis was performed using two‐way ANOVA followed by Tukey's post‐test. *p < 0.05, ***p < 0.001 and ****p < 0.0001.

**FIGURE 4:**
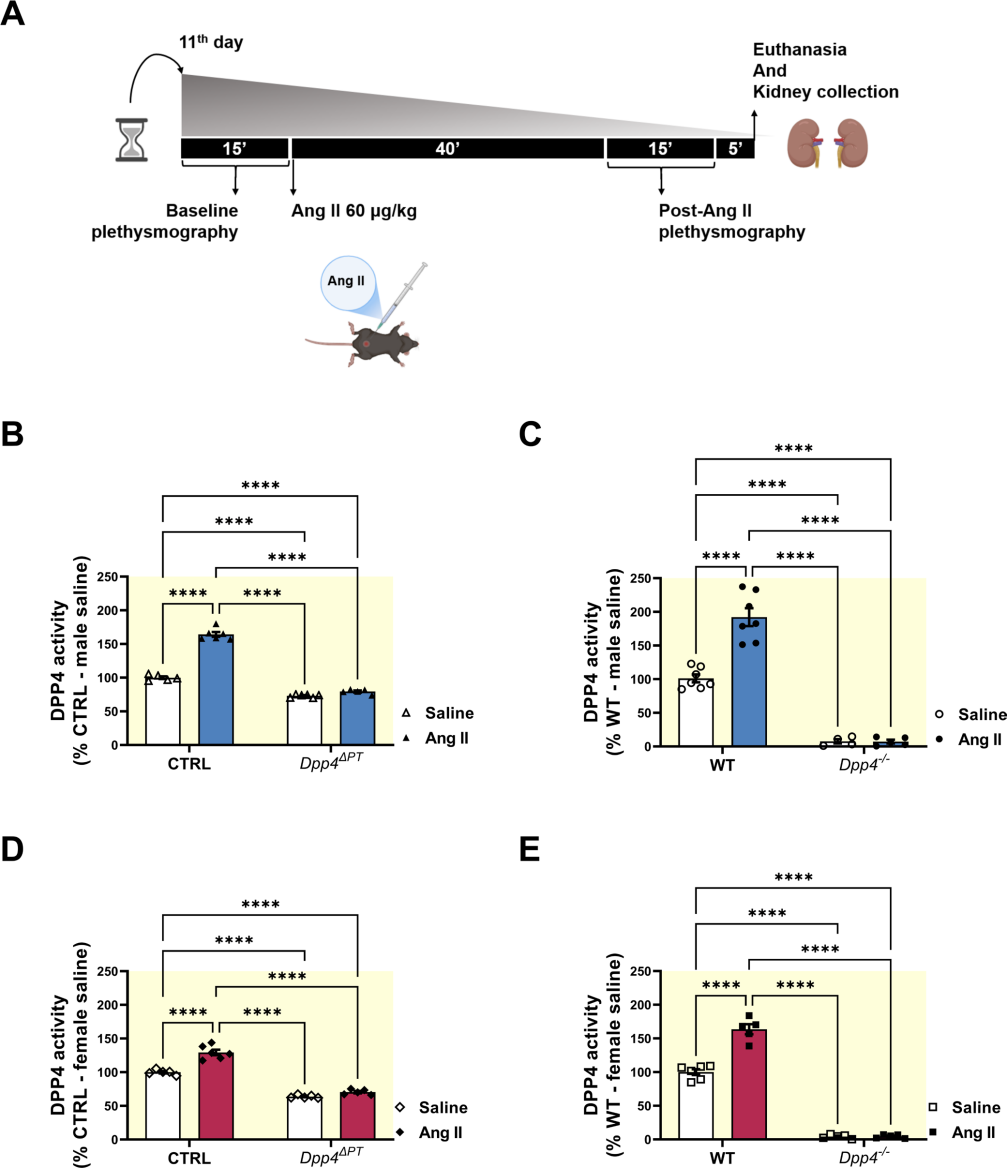
Effect of acute Ang II administration on the renal DPP4 activity of male and female mice. (A) Experimental design of the acute Ang II injection protocol. (B–E) Renal DPP4 activity was assessed by fluorimetry in renal homogenates from male and female (B, D) *Dpp4* ^ΔPT^ and (C, E) *Dpp4* ^−/−^ mice. Each dot represents the % of DPP4 activity relative to CTRL or WT per animal. Bars represent mean ± SEM. Data normality was assessed with the Shapiro–Wilk test. The experimental n ranged from 4 to 9. Statistical analysis was performed using two‐way ANOVA followed by Tukey's post‐test. ****p < 0.0001.

**FIGURE 5:**
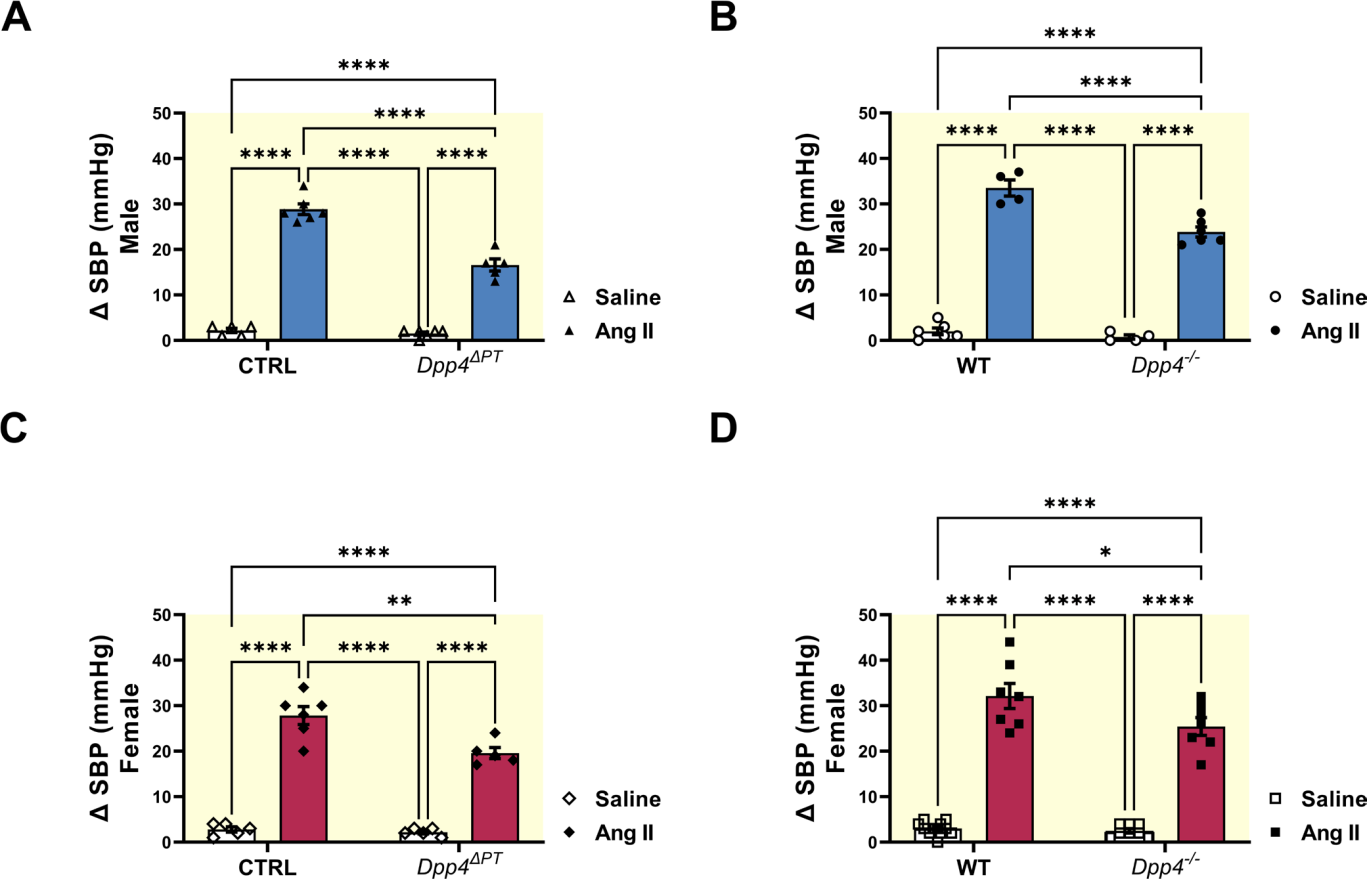
Effect of a pressor dose of Ang II on blood pressure in *Dpp4* ^ΔPT^ and *Dpp4* ^−/−^ mice. Systolic blood pressure (SBP) was measured by tail‐cuff plethysmography before and after Ang II administration in male and female (A, B) *Dpp4* ^ΔPT^ and (C, D) *Dpp4* ^−/−^mice. Each dot represents the ΔSBP change per animal. Bars represent mean ± SEM. Data normality was assessed with the Shapiro–Wilk test. The experimental n ranged from 4 to 11. Statistical analysis was performed using two‐way ANOVA followed by Tukey's post‐test. *p < 0.05; **p < 0.01; and ****p < 0.0001.

**FIGURE 6:**
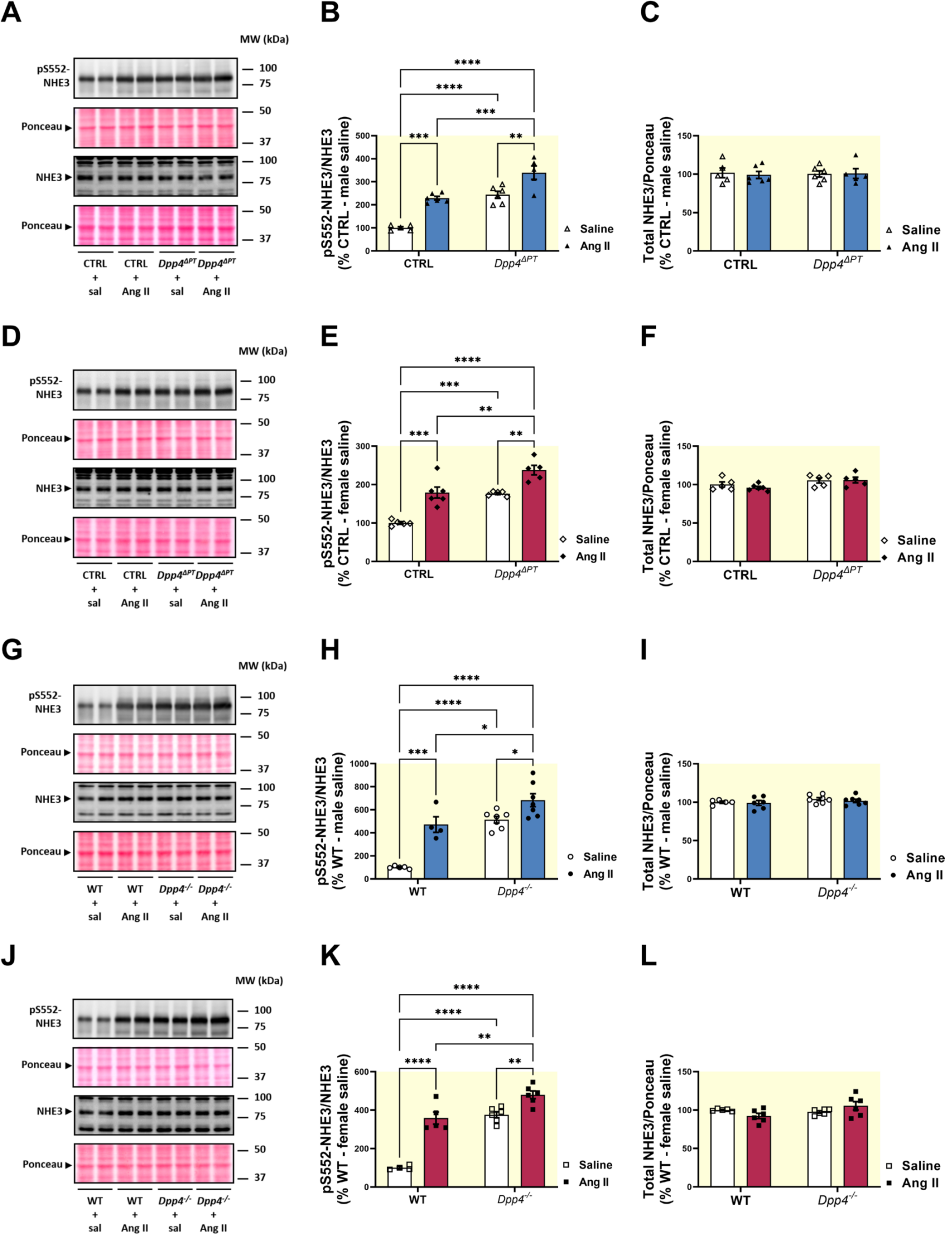
Influence of acute Ang II‐induced blood pressure rise on NHE3 phosphorylation in the kidneys of *Dpp4* ^ΔPT^ and *Dpp4* ^−/−^ mice. Levels of phosphorylated (pS552‐NHE3) and total NHE3 were determined by immunoblotting in kidney homogenates from *Dpp4* ^ΔPT^ (A–F) and *Dpp4* ^−/−^ (G–L) mice. Data normalized to Ponceau staining. Each dot represents the % of pS552‐NHE3/NHE3 relative to CTRL or WT per animal. Bars represent mean ± SEM. Data normality was assessed with the Shapiro–Wilk test. The experimental n ranged from 4 to 7. Statistical analysis was performed using two‐way ANOVA followed by Tukey's post‐test. *p < 0.05, **p < 0.01, ***p < 0.001 and ****p < 0.0001.

Data are presented as mean ± standard error of the mean (SEM). The sample size (n) for each analysis is indicated by individual points in the scatter‐dot plots. Power analyses were performed with the G*Power 3.1 software [39] (Table S9). Statistical analyses were performed using GraphPad Prism 10.0 (San Diego, CA). Normality was assessed with the Shapiro–Wilk test. Group comparisons were conducted using Student's *t*‐test for two groups (Figures S5 and S8–S10), and one‐way (Figure S4) or two‐way ANOVA (Figures 1, 2, 3, 4, 5, 6 and Figures S6 and S7) with Tukey's post hoc test for four or more groups. Statistical significance was set at *p* < 0.05.

## 2 Results

### 2.1 Phenotypic Characterization of PT‐Specific *Dpp4* Deletion in Mice

Mice with PT‐specific deletion of *Dpp4* (*Dpp4* ^ΔPT^) showed a ~35% reduction in kidney DPP4 in males and a ~45% reduction in females compared to CTRL mice (Figure 1A). Immunostaining of kidney sections for DPP4 and SGLT2, a PT marker, confirmed that this reduction was specific to the PT. In CTRL mice, DPP4 is evidenced in both the PT, where it colocalizes with SGLT2, and the glomeruli. In contrast, *Dpp4* ^ΔPT^ mice showed DPP4 staining exclusively in the glomeruli (Figure 1C). Similarly, kidney DPP4 activity decreased by approximately 30% in *Dpp4* ^ΔPT^ males and 40% in *Dpp4* ^ΔPT^ females compared to CTRL mice (Figure 1E). Mice with global *Dpp4* deletion (*Dpp4* ^−/−^) showed absence of DPP4 protein (Figure 1B), staining (Figure 1D), and activity (Figure 1F). Consistent with previous findings [35], kidney DPP4 exhibited sexual dimorphism, with higher abundance and activity in females than in males (Figure 1).

SBP assessment by plethysmography showed no baseline differences between *Dpp4* ^ΔPT^ and CTRL (Figure 2A) or between *Dpp4* ^−/−^ and WT mice (Figure 2B), with preserved sex‐based BP differences, as female *Dpp4* ^ΔPT^ exhibited lower BP than males. Despite comparable SBP, both *Dpp4* ^ΔPT^ and *Dpp4* ^−/−^ mice exhibited more rapid acute diuretic (Figure 2C,D) and natriuretic (Figure 2E,F) responses to a saline challenge compared to littermate controls. Consistent with previous evidence [35], acute diuretic (Figure 2C,D) and natriuretic (Figure 2E,F) responses to a saline load were faster in female mice than in males. Interestingly, mice with *Dpp4* deletion (both PT‐specific and global) exhibited comparable fluid and salt excretion percentages between males and females (Figure 2C–F).

The more rapid diuretic and natriuretic responses to a saline challenge in *Dpp4* ^ΔPT^ mice suggest reduced sodium and fluid reabsorption in the PT, a function primarily mediated by NHE3. Given that some studies have linked DPP4 inhibition to downregulation of NHE3 activity and increased pS552‐NHE3 levels [40], we investigated kidney pS552‐NHE3 levels in our experimental models. As previously reported, females had higher renal pS552‐NHE3 levels than males [35]. Notably, pS552‐NHE3 levels were approximately twofold higher in *Dpp4* ^ΔPT^ mice (Figure 3A,B) and fourfold higher in *Dpp4* ^−/−^ mice, in both males and females, compared to their respective controls (Figure 3D,E). The greater increase in pS552‐NHE3 in *Dpp4* ^−/−^ mice compared to *Dpp4* ^ΔPT^ mice may be partly due to background differences between CTRL (*Dpp4* ^Fl/Fl^) and WT mice, as CTRL mice exhibited higher renal pS552‐NHE3 levels than WT (Figure S5). Consequently, the difference between *Dpp4* ^ΔPT^ and *Dpp4* ^Fl/Fl^ was less pronounced than between *Dpp4* ^−/−^ and WT.

The sexual dimorphism in pS552‐NHE3 was preserved in the absence of DPP4, being predominantly higher in females than in their male counterparts (Figure 3A,B,D,E). The total NHE3 abundance remained unchanged across all experimental groups (Figure 3C,F), consistent with previous findings showing that DPP4 influences NHE3 through posttranslational mechanisms rather than altering its abundance [22, 40].

Because both the *Dpp4* ^ΔPT^ and *Dpp4* ^−/−^ mice are constitutive models, adaptive mechanisms may develop over time to compensate for the absence of DPP4, resulting in unchanged baseline BP compared to their respective controls (Figure 2A,B). To determine whether compensatory mechanisms in the distal nephron contribute to BP maintenance in these models, we analyzed total sodium‐chloride cotransporter (NCC) abundance, its phosphorylated active form at threonine 53 (pNCC) [41], and the abundance of full‐length and cleaved epithelial sodium channel (ENaC) subunits α and γ in kidneys from male mice. In *Dpp4* ^ΔPT^ males, pNCC was significantly higher than in CTRL mice (169% ± 14% vs. 100% ± 17%, *p* < 0.05), whereas total NCC abundance remained unchanged (Figure S6A,C). In contrast, *Dpp4* ^−/−^ males exhibited a marked upregulation of both pNCC (310% ± 28% vs. 100% ± 8%, *p* < 0.001) and total NCC (162% ± 11% vs. 100% ± 4%, *p* < 0.01), compared to WT littermates (Figure S6B,D). We next examined ENaC. In *Dpp4* ^ΔPT^ mice, neither full‐length nor cleaved (active) α‐ and γ‐ENaC subunits differed from CTRL (Figure S7A,B,E,F). By contrast, *Dpp4* ^−/−^ males displayed significantly higher levels of cleaved α‐ENaC and cleaved γ‐ENaC than WT mice, while the abundance of full‐length forms remained unchanged (Figure S7C,D,G,H).

### 2.2 Acute Ang II‐Induced BP Elevation Is Attenuated in Both *Dpp4* ^ΔPT^ and *Dpp4* ^−/−^ Mice

To assess the contribution of PT DPP4 to BP regulation, we used infusion of Ang II [32], a potent vasoconstrictor, as a physiological challenge to test whether PT DPP4 is required for a full pressor response. More specifically, we assessed whether the BP increase following Ang II injection was attenuated in *Dpp4* ^ΔPT^ mice compared to CTRL, and whether global *Dpp4* deletion produced a similar or greater blunting of the Ang II‐induced pressor response, thereby providing insight into the relative contribution of PT versus systemic DPP4.

Although Ang II is not a substrate for DPP4, our previous work showed that it stimulates PT DPP4 activity [42]. Accordingly, as shown in Figure 4, CTRL and WT mice treated with a pressor dose of Ang II (60 μg/kg) [33] showed a higher DPP4 activity, with an approximate increase of 50% in males and 30% in females compared to saline. Interestingly, residual kidney DPP4 activity in *Dpp4* ^ΔPT^ mice remained unchanged in response to Ang II (Figure 4B,D), suggesting that Ang II specifically regulates PT DPP4 activity. Total DPP4 abundance remained unchanged under both saline and Ang II conditions in WT mice (Figure S8).

SBP was measured before and after Ang II injection (Figure S9), and the change in BP (ΔSBP) was calculated. Ang II administration increased SBP across all experimental groups (Figure S9, right panels). As seen in Figure 5, the acute pressor response (ΔSBP = Post‐Ang II SBP—Baseline BP) was significantly attenuated in *Dpp4* ^ΔPT^ compared to CTRL males: 17 ± 1 vs. 29 ± 1 mmHg (*p* < 0.0001) and females: 20 ± 1 vs. 28 ± 2 mmHg (*p* < 0.002). Similarly, ΔSBP was also lower in *Dpp4* ^−/−^ mice compared to WT males: 24 ± 1 vs. 34 ± 2 mmHg (*p* < 0.0001) and females: 25 ± 2 vs. 32 ± 3 mmHg (*p* < 0.03), demonstrating that PT DPP4 contributes to the acute pressor response to Ang II independently of sex.

Next, we investigated whether the reduced acute pressor response to Ang II was associated with further upregulation of pS552‐NHE3 in *Dpp4* ^ΔPT^ and *Dpp4* ^−/−^ mice (Figure 6). In CTRL mice, an acute pressor dose of Ang II significantly increased pS552‐NHE3 levels (males: Ang II, 229% ± 8% vs. saline, 100% ± 5%, *p* < 0.0002; females: Ang II, 180% ± 14% vs. saline, 100% ± 3%, *p* < 0.0002). In *Dpp4* ^ΔPT^ mice, however, Ang II further increased pS552‐NHE3 by 95% in males and 61% in females (Figure 6A,B,D,E). Similar findings were observed in *Dpp4* ^*−/−*^ mice. Ang II increased WT pS552‐NHE3 levels (males: Ang II 472% ± 68% vs. saline 100% ± 5%, *p* < 0.0005; females: Ang II 359 ± 32 vs. saline 100 ± 6%, *p* < 0.0001). In contrast, Ang II injection in *Dpp4* ^−/−^ mice resulted in a greater increase in pS552‐NHE3 (176% in males and 104% in females) (Figure 6G,H,J,K). Total NHE3 levels remained constant across all experimental conditions (Figure 6C,F,I,L). These findings suggest that the absence of DPP4 enhances pS552‐NHE3, thereby attenuating the acute pressor response to Ang II.

## 3 Discussion

This study is the first to examine the impact of PT‐specific *Dpp4* deletion on natriuresis, renal pS552‐NHE3 levels, and BP. Our findings demonstrate that both PT‐specific and global *Dpp4* knockout models similarly enhance mice's natriuretic and diuretic responses to saline load. These findings highlight DPP4's role in mechanisms regulating salt reabsorption, likely within the PT. Furthermore, both *Dpp4* ^ΔPT^ and *Dpp4* ^−/−^ mice exhibit upregulation of renal pS552‐NHE3 levels, suggesting a baseline reduction in PT NHE3 activity. The comparable reduction in the Ang II‐induced BP rise observed in both knockout models, relative to littermate controls, suggests that PT‐specific *Dpp4* deletion uniquely counteracts the acute pressor effect of Ang II, likely by enhancing the pressure‐natriuresis response.

We previously demonstrated that DPP4 preferentially interacts with NHE3 in the body of the microvilli [3], where NHE3 is active [43, 44], while phosphorylated NHE3 at serine 552 (pS552‐NHE3) localizes to the base of the brush‐border microvilli [45], where NHE3 is inactive [43, 44]. In this study, we found that pS552‐NHE3 levels are significantly higher in *Dpp4* knockout mice than in controls, supporting the notion that baseline NHE3 activity is reduced in the absence of DPP4. These findings raised two key questions: (i) Why does *Dpp4* deletion enhance NHE3 phosphorylation? (ii) Is DPP4 involved in regulating NHE3's subcellular distribution? As serine 552 (S552) is a consensus site for protein kinase A (PKA)‐mediated inhibition of NHE3 [46], one plausible mechanism for increased pS552‐NHE3 levels following *Dpp4* deletion is the enhanced bioavailability of DPP4 substrates such as glucagon‐like peptide‐1 (GLP‐1), which activates Gs‐coupled receptors [47]. GLP‐1 is known to promote natriuresis, at least in part, through PKA‐dependent inhibition of NHE3 via pS552 phosphorylation. However, the natriuretic effects of DPP4is are also observed in mice lacking the GLP‐1R [21] and in isolated PT cells [20] that do not produce GLP‐1. These findings suggest that DPP4's regulation of NHE3 activity and phosphorylation may also occur independently of GLP‐1, potentially involving alternative signaling pathways or protein interactions. In this regard, we have previously demonstrated that the interaction between DPP4 and NHE3 is indirect and requires intermediary proteins [48]. Among these, motor proteins involved in NHE3's subcellular distribution across brush‐border microdomains are likely candidates [49]. Ongoing studies aim to clarify these mechanisms and identify additional mediators of the DPP4‐NHE3 interaction.

Our data show that female mice exhibit higher DPP4 expression and enzymatic activity, consistent with findings in rats and humans [35, 50, 51]. Despite DPP4's role in stimulating NHE3 activity, females paradoxically have higher pS552‐NHE3 levels and a faster natriuretic response to saline challenge than males. This discrepancy could be explained by a lower expression of intermediary proteins mediating the DPP4‐NHE3 interaction in females, which may reduce NHE3 activation despite elevated DPP4 levels.

Despite elevated renal pS552‐NHE3 levels in both *Dpp4* knockout models, baseline SBP remained unchanged compared to controls, suggesting compensatory adjustments in sodium handling downstream of the proximal tubule. A likely contributor is the sodium‐chloride cotransporter (NCC), which we previously reported to be upregulated in the distal convoluted tubule in response to proximal NHE3 inhibition by sodium‐glucose cotransporter‐2 inhibitors (SGLT2i) in normotensive rats [36]. Consistent with this, we now show that NCC phosphorylation at threonine 53 (pNCC), the active form of the transporter, is upregulated in both PT‐specific and global *Dpp4* knockout mice. Notably, in global *knockouts*, compensation extended beyond pNCC, with significant upregulation of total NCC and activation of ENaC subunits, suggesting that broader recruitment of distal transport pathways is required to preserve BP in the absence of systemic DPP4. In contrast, in male *Dpp4* ^ΔPT^ mice, enhanced NCC phosphorylation alone appears sufficient.

Our findings also demonstrate that the acute Ang II‐mediated BP rise was significantly attenuated in both *Dpp4* ^ΔPT^ and *Dpp4* ^−/−^. This attenuation was accompanied by further upregulation of kidney NHE3 phosphorylation at serine 552. Elevated kidney pS552‐NHE3 and NHE3 redistribution within microvillar microdomains, resulting in reduced NHE3 activity, have been associated with pressure‐natriuresis in several hypertension models [14, 17, 52, 53]. In SHRs, for instance, PT NHE3‐mediated sodium reabsorption is higher before hypertension onset but subsequently declines compared to normotensive rats [14]. In the pre‐hypertensive phase, SHRs show a higher abundance of NHE3 in the body of the microvilli, where it is associated with DPP4 and lower pS552‐NHE3 levels. Once hypertension is established, however, this association is reduced, and pS552‐NHE3 is higher, diminishing PT sodium reabsorption and contributing to pressure‐natriuresis [14]. Similarly, DPP4is attenuate BP in pre‐hypertensive SHRs but lose their effectiveness once hypertension is established [25]. A similar pattern is seen in Ang II‐induced hypertension, where DPP4is fail to lower BP after hypertension onset [27]. These observations suggest that one plausible explanation for the conflicting data on the effects of DPP4is on BP is that their ability to enhance pS552‐NHE3 levels and inhibit NHE3 activity is already maximized in established hypertension, rendering further intervention ineffective. Furthermore, as DPP4is and RAS blockers share overlapping mechanisms [38, 54, 55], their combined use in hypertension therapy warrants further investigation, as it may amplify adverse effects.

Accumulating evidence from our group and others highlights a crosstalk between the signaling pathways activated by Ang II/AT1R and DPP4 [55]. In cultured PT cells, supraphysiological concentrations of Ang II enhance DPP4 activity in an ERK 1/2‐dependent manner through AT1R activation [56]. Conversely, DPP4is prevent Ang II/AT1R‐mediated activation of ERK 1/2. Consistent with these observations, we found that the Ang II‐induced increase in DPP4 activity is confined to PT DPP4, as kidney DPP4 activity remained unchanged in *Dpp4* ^ΔPT^ mice following Ang II treatment. Interestingly, renal Ang II levels were elevated in both *Dpp4* ^ΔPT^ and *Dpp4* ^−/−^, whereas *AT1R*‐mRNA expression was increased only in the global knockout compared to respective controls (Figure S10), potentially reflecting a compensatory response to impaired downstream signaling. Importantly, we have previously shown that the interaction between Ang II/AT1R and DPP4 is pivotal in the pathophysiology of kidney diseases, with DPP4 inhibition preventing glomerular and tubulointerstitial injury, proteinuria, oxidative stress, inflammation, and fibrosis [24, 56, 57, 58] processes that are at least partially driven by Ang II/AT1R signaling. Our current findings expand the understanding of the Ang II/AT1R‐DPP4 crosstalk, suggesting that it plays a critical role not only in kidney disease pathophysiology but also in proximal tubular function.

In summary, our findings suggest that PT DPP4 exerts an anti‐natriuretic effect by tonically stimulating NHE3 through signaling pathways that prevent phosphorylation of serine 552, a key residue associated with the inhibition of PT NHE3‐mediated sodium reabsorption. In the absence of DPP4, these regulatory mechanisms are altered, leading to sustained upregulation of pS552‐NHE3 levels and reduced BP sensitivity to Ang II, likely due to an enhanced pressure–natriuresis response. Further studies are needed to identify the signaling pathways activated by DPP4 under physiological conditions, as well as their potential impact on NHE3 regulation and other proximal tubular functions.

## Disclosure

The authors have nothing to report.

## Conflicts of Interest

The authors declare no conflicts of interest.

## Supporting information


**Data S1:** apha70127‐sup‐0001‐Supinfo.docx.

## Data Availability

The authors confirm that the data supporting the findings of this study is available within the article and its Data S1.
