# Supplementary material for: Unique Role of Proximal Tubule Dipeptidyl Peptidase 4 on Blood Pressure, Renal Sodium Handling, and Na+/H+ Exchanger Isoform 3 Phosphorylation
Source: Acta Physiol (Oxf). 2025 Nov 4;241(12):e70127. doi: 10.1111/apha.70127 (PMC12598389; doi:10.1111/apha.70127)
Supplement: Supplementary file 1 — Data S1: apha70127‐sup‐0001‐Supinfo.docx. [file APHA-241-e70127-s001.docx]

**SUPPLEMENTAL MATERIAL**

**Unique Role of Proximal Tubule Dipeptidyl Peptidase 4 on Blood Pressure, Renal Sodium Handling, and Na⁺/H⁺ Exchanger Isoform 3 Phosphorylation**

Flavia L. Martins PhD^1,2*^, Joao Carlos Ribeiro-Silva PhD^3*^, Érika F. Jesus^1^, Ravi Nistala MD^2^, and Adriana C. C. Girardi PhD^1^

^1^Departamento de Cardiopneumologia, Universidade de São Paulo, São Paulo, SP, Brazil; ^2^Division of Nephrology, Department of Medicine, University of Missouri School of Medicine, Columbia, MO, USA; ^3^State University of New York (SUNY) Upstate Medical University, Syracuse, NY, USA

*These authors contributed equally to this work and should both be regarded as first authors.

**Supplementary Table S1.** **Genotyping conditions.**

| **Reaction** | **Primer sequence** | **PCR conditions** |
| --- | --- | --- |
| *Dpp4*^-/-^ | Forward 5’ – GAATATGATCCTTGTCAGAGCAGCC - 3’  Reverse 5’ – CTGCACTCAGAAGTCTCACTG – 3’  Control primer  Forward 5' – GAGACTCTGGCTACTCATCC – 3'  Reverse 5' – CCTTCAGCAAGAGCTGGGGAC – 3' | Step 1: 95°C, 15 min Step 2: 94°C, 45 secs Step 3: 60°C, 1 min Step 4: 72°C, 1min  (37 cycles – Steps 2-4) Step 5: 72°C 5min  Cooling 4°C ∞ |
| *Dpp4^Fl/Fl^* | Forward 5’ – GGAGGGTGAATTTATAATCCTTTACC - 3’  Reverse 5’ – CTGCACTCAGAAGTCTCACTG – 3’ | Step 1: 95°C, 15 min Step 2: 94°C, 45 secs Step 3: 60°C, 1 min Step 4: 72°C, 1min  (35 cycles – Steps 2-4) Step 5: 72°C 5min  Cooling 4°C ∞ |
| *Cre* | Forward 5' – CCTGGAAAATGCTTCTGTCCG- 3'  Reverse 5' – CAGGGTGTTATAAGCAATCCC – 3' | Step 1: 95°C, 15 min Step 2: 94°C, 45 secs Step 3: 60°C, 1 min Step 4: 72°C, 1min  (35 cycles – Steps 2-4) Step 5: 72°C 5min  Cooling 4°C ∞ |

**Supplementary Table S2. Raw systolic blood pressure measurements for WT and *Dpp4*^-/-^ male mice**

| **MALES** |  | **Genotype** | **IP injection** | **Mice** | **Measurement** | **Systolic Blood Pressure Measurements** | | | | | | | | | | **Average** |
| --- | --- | --- | --- | --- | --- | --- | --- | --- | --- | --- | --- | --- | --- | --- | --- | --- |
|  |  |  |  |  |  | **1** | **2** | **3** | **4** | **5** | **6** | **7** | **8** | **9** | **10** |  |
|  |  | **WT** | Saline | 1 | Pre | 106 | 103 | 108 | 108 | 107 | 107 | 108 | 106 | 108 | 110 | 107 |
|  |  |  |  |  | Post | 105 | 101 | 113 | 106 | 104 | 118 | 102 | 102 | 103 | 101 | 106 |
|  |  |  |  | 2 | Pre | 113 | 113 | 112 | 114 | 117 | 120 | 122 | 107 | 114 | 109 | 114 |
|  |  |  |  |  | Post | ND | | | | | | | | | | |
|  |  |  |  | 3 | Pre | 122 | 122 | 136 | 113 | 105 | 108 | 110 | 109 | 115 | 107 | 115 |
|  |  |  |  |  | Post | 118 | 113 | 108 | 119 | 110 | 112 | 115 | 113 | 120 | 119 | 115 |
|  |  |  |  | 4 | Pre | 107 | 108 | 103 | 98 | 101 | 100 | 106 | 106 | 108 | 106 | 104 |
|  |  |  |  |  | Post | 95 | 100 | 100 | 97 | 105 | 120 | 115 | 103 | 103 | 103 | 104 |
|  |  |  |  | 5 | Pre | 101 | 121 | 105 | 108 | 110 | 102 | 111 | 112 |  |  | 109 |
|  |  |  |  |  | Post | 120 | 111 | 109 | 115 | 108 | 114 | 111 | 118 |  |  | 113 |
|  |  |  |  | 6 | Pre | 104 | 116 | 109 | 116 | 109 | 109 | 110 | 101 | 107 | 110 | 109 |
|  |  |  |  |  | Post | 109 | 110 | 110 | 115 | 106 | 108 | 110 | 123 |  |  | 111 |
|  |  |  |  | 7 | Pre | 100 | 129 | 109 | 110 | 105 | 106 | 109 | 126 |  |  | 112 |
|  |  |  |  |  | Post | 94 | 105 | 117 | 118 | 115 | 110 | 115 | 105 | 116 |  | 111 |
|  |  |  | Ang II | 8 | Pre | 122 | 105 | 113 | 113 | 102 | 108 | 121 | 112 | 110 | 106 | 111 |
|  |  |  |  |  | Post | 144 | 152 | 158 | 152 | 159 | 152 | 141 | 138 | 153 | 137 | 149 |
|  |  |  |  | 9 | Pre | 112 | 108 | 102 | 105 | 104 | 110 | 109 | 99 | 115 | 99 | 106 |
|  |  |  |  |  | Post | ND | | | | | | | | | | |
|  |  |  |  | 10 | Pre | 118 | 115 | 114 | 116 | 108 | 107 | 108 | 111 |  |  | 112 |
|  |  |  |  |  | Post | 156 | 144 | 132 | 136 | 147 | 139 | 138 | 149 | 151 |  | 144 |
|  |  |  |  | 11 | Pre | 121 | 122 | 116 | 118 | 109 | 115 | 116 | 120 | 113 | 105 | 116 |
|  |  |  |  |  | Post | 157 | 174 | 160 | 148 | 155 | 148 | 141 | 132 | 140 | 156 | 151 |
|  |  |  |  | 12 | Pre | 109 | 105 | 110 | 116 | 113 | 103 | 110 | 114 | 110 |  | 110 |
|  |  |  |  |  | Post | 147 | 138 | 135 | 139 | 149 | 140 | 140 | 136 | 141 | 135 | 140 |
|  |  | ***Dpp4^-/-^*** | Saline | 13 | Pre | 109 | 110 | 107 | 105 | 105 | 109 | 103 | 116 | 110 | 110 | 108 |
|  |  |  |  |  | Post | 109 | 115 | 115 | 110 | 116 | 105 | 105 | 105 | 108 | 106 | 109 |
|  |  |  |  | 14 | Pre | 117 | 117 | 110 | 105 | 102 | 108 | 102 | 115 | 118 | 121 | 112 |
|  |  |  |  |  | Post | ND | | | | | | | | | | |
|  |  |  |  | 15 | Pre | 110 | 108 | 106 | 111 | 108 | 110 | 115 | 109 | 107 |  | 109 |
|  |  |  |  |  | Post | 126 | 112 | 110 | 109 | 106 | 107 | 111 | 105 | 112 |  | 111 |
|  |  |  |  | 16 | Pre | 106 | 105 | 111 | 103 | 109 | 103 | 106 | 118 | 122 |  | 109 |
|  |  |  |  |  | Post | 115 | 107 | 111 | 112 | 110 | 111 | 108 | 107 | 106 |  | 110 |
|  |  |  |  | 17 | Pre | 113 | 106 | 119 | 108 | 123 | 118 | 105 | 107 |  |  | 112 |
|  |  |  |  |  | Post | ND | | | | | | | | | | |
|  |  |  |  | 18 | Pre | 111 | 108 | 112 | 108 | 102 | 112 | 110 | 107 |  |  | 109 |
|  |  |  |  |  | Post | ND | | | | | | | | | | |
|  |  |  |  | 19 | Pre | 109 | 105 | 123 | 114 | 99 | 109 | 107 |  |  |  | 109 |
|  |  |  |  |  | Post | 110 | 110 | 114 | 127 | 119 | 106 | 101 | 96 | 101 | 108 | 109 |
|  |  |  | Ang II | 20 | Pre | 123 | 117 | 123 | 121 | 101 | 102 | 102 | 94 | 123 | 103 | 111 |
|  |  |  |  |  | Post | ND | | | | | | | | | | |
|  |  |  |  | 21 | Pre | 116 | 125 | 121 | 120 | 126 | 119 | 110 | 110 | 110 | 119 | 118 |
|  |  |  |  |  | Post | 141 | 147 | 146 | 142 | 147 | 139 | 148 | 148 | 143 | 142 | 144 |
|  |  |  |  | 22 | Pre | 108 | 101 | 102 | 122 | 118 | 110 | 122 | 112 | 106 | 102 | 110 |
|  |  |  |  |  | Post | 142 | 136 | 143 | 132 | 143 | 141 | 137 | 135 | 132 | 131 | 137 |
|  |  |  |  | 23 | Pre | 122 | 113 | 117 | 115 | 111 | 106 | 113 | 109 |  |  | 113 |
|  |  |  |  |  | Post | ND | | | | | | | | | | |
|  |  |  |  | 24 | Pre | 108 | 111 | 115 | 119 | 120 | 113 | 105 | 108 |  |  | 112 |
|  |  |  |  |  | Post | ND | | | | | | | | | |  |
|  |  |  |  | 25 | Pre | 111 | 101 | 102 | 110 | 113 | 119 | 106 | 108 | 105 |  | 108 |
|  |  |  |  |  | Post | 138 | 134 | 124 | 122 | 136 | 135 | 129 | 127 |  |  | 131 |
|  |  |  |  | 26 | Pre | 114 | 115 | 111 | 104 | 106 | 115 | 114 | 118 | 116 |  | 113 |
|  |  |  |  |  | Post | 145 | 130 | 129 | 135 | 132 | 134 | 129 | 129 | 135 |  | 133 |
|  |  |  |  | 27 | Pre | 109 | 111 | 110 | 111 | 101 | 109 | 107 | 112 | 113 |  | 109 |
|  |  |  |  |  | Post | 131 | 128 | 131 | 133 | 133 | 135 | 134 | 133 | 138 |  | 133 |
|  |  |  |  | 28 | Pre | 101 | 119 | 110 | 105 | 119 | 116 | 103 | 115 | 102 |  | 110 |
|  |  |  |  |  | Post | 149 | 129 | 128 | 121 | 126 | 124 | 130 | 135 | 129 | 144 | 132 |

ND: not detectable; PA values could not be acquired.

**Supplementary Table S3. Raw systolic blood pressure measurements for WT and *Dpp4*^-/-^ female mice**

| **FEMALES** | **Genotype** | **IP injection** | **Mice** | **Measurement** | **Systolic Blood Pressure Measurements** | | | | | | | | | | **Average** |
| --- | --- | --- | --- | --- | --- | --- | --- | --- | --- | --- | --- | --- | --- | --- | --- |
|  |  |  |  |  | **1** | **2** | **3** | **4** | **5** | **6** | **7** | **8** | **9** | **10** |  |
|  | **WT** | Saline | 1 | Pre | 100 | 101 | 100 | 101 | 99 | 104 | 100 | 102 | 99 | 95 | 100 |
|  |  |  |  | Post | 105 | 106 | 99 | 98 | 100 | 101 | 101 | 99 | 108 |  | 102 |
|  |  |  | 2 | Pre | 78 | 83 | 103 | 98 | 101 | 88 | 98 | 101 | 102 | 88 | 94 |
|  |  |  |  | Post | 103 | 105 | 110 | 99 | 98 | 87 | 91 | 96 |  |  | 99 |
|  |  |  | 3 | Pre | 107 | 108 | 105 | 105 | 102 | 101 | 102 | 101 |  |  | 104 |
|  |  |  |  | Post | 112 | 115 | 106 | 103 | 104 | 102 | 99 | 101 | 109 |  | 106 |
|  |  |  | 4 | Pre | 105 | 98 | 95 | 93 | 104 | 105 | 89 | 94 | 96 |  | 98 |
|  |  |  |  | Post | 108 | 110 | 101 | 98 | 99 | 97 | 109 | 101 | 99 | 89 | 101 |
|  |  |  | 5 | Pre | 89 | 92 | 95 | 97 | 92 | 95 | 88 | 93 | 89 | 98 | 93 |
|  |  |  |  | Post | 100 | 101 | 95 | 88 | 93 | 103 | 98 | 98 | 98 | 89 | 96 |
|  |  |  | 6 | Pre | 104 | 98 | 99 | 98 | 100 | 94 | 89 | 98 | 89 | 98 | 97 |
|  |  |  |  | Post | 96 | 95 | 96 | 95 | 94 | 91 | 90 | 96 | 90 | 96 | 94 |
|  |  |  | 7 | Pre | 104 | 92 | 93 | 101 | 101 | 88 | 93 | 95 | 99 |  | 96 |
|  |  |  |  | Post | 101 | 105 | 102 | 103 | 110 | 99 | 98 | 96 | 92 | 99 | 101 |
|  |  |  | 8 | Pre | 108 | 113 | 111 | 118 | 118 | 116 | 104 | 115 | 113 | 114 | 113 |
|  |  |  |  | Post | 110 | 113 | 114 | 113 | 111 | 110 | 116 | 117 | 111 | 115 | 113 |
|  |  |  | 9 | Pre | 98 | 95 | 88 | 87 | 83 | 85 | 98 | 99 |  |  | 92 |
|  |  |  |  | Post | 80 | 82 | 82 | 88 | 98 | 88 | 85 | 87 |  |  | 86 |
|  |  |  | 10 | Pre | 98 | 88 | 83 | 83 | 85 | 80 | 92 | 85 | 88 | 84 | 87 |
|  |  |  |  | Post | 95 | 85 | 92 | 92 | 82 | 92 | 91 | 95 |  |  | 91 |
|  |  |  | 11 | Pre | 104 | 105 | 101 | 106 | 108 | 102 | 109 | 100 | 99 | 99 | 103 |
|  |  |  |  | Post | 112 | 102 | 102 | 110 | 113 | 102 | 110 | 99 | 100 | 101 | 105 |
|  |  | Ang II | 12 | Pre | 88 | 91 | 97 | 93 | 90 | 88 | 88 | 83 | 88 | 87 | 89 |
|  |  |  |  | Post | 121 | 107 | 112 | 128 | 118 | 122 | 117 | 115 | 108 | 115 | 116 |
|  |  |  | 13 | Pre | 89 | 87 | 91 | 90 | 91 | 85 | 91 | 95 | 86 | 86 | 89 |
|  |  |  |  | Post | 110 | 113 | 110 | 112 | 115 | 126 | 110 | 117 | 110 | 104 | 113 |
|  |  |  | 14 | Pre | 115 | 117 | 113 | 118 | 124 | 126 | 119 | 125 | 119 | 118 | 119 |
|  |  |  |  | Post | 84 | 90 | 82 | 92 | 86 | 89 | 90 | 116 | 95 | 92 | 92 |
|  |  |  | 15 | Pre | 100 | 99 | 104 | 103 | 73 | 98 | 99 | 100 | 94 |  | 97 |
|  |  |  |  | Post | 142 | 129 | 130 | 131 | 129 | 137 | 144 | 125 | 139 | 145 | 135 |
|  |  |  | 16 | Pre | 96 | 95 | 91 | 99 | 100 | 95 | 95 | 99 | 97 | 95 | 96 |
|  |  |  |  | Post | 121 | 118 | 125 | 123 | 127 | 117 | 124 | 129 | 125 | 123 | 123 |
|  |  |  | 17 | Pre | 106 | 108 | 102 | 112 | 103 | 114 | 101 | 110 | 104 |  | 107 |
|  |  |  |  | Post | 134 | 136 | 141 | 136 | 141 | 137 | 135 | 136 | 154 | 139 | 139 |
|  |  |  | 18 | Pre | 111 | 108 | 118 | 112 | 113 | 116 | 115 | 105 |  |  | 112 |
|  |  |  |  | Post | 166 | 167 | 149 | 142 | 164 | 157 | 150 | 162 | 148 |  | 156 |
|  |  |  | 19 | Pre | 106 | 108 | 114 | 105 | 105 | 109 | 103 | 112 | 111 | 101 | 107 |
|  |  |  |  | Post | ND | | | | | | | | | | |
|  |  |  | 20 | Pre | 87 | 94 | 99 | 96 | 101 | 95 | 98 | 97 | 89 | 88 | 94 |
|  |  |  |  | Post | 126 | 133 | 124 | 121 | 122 | 126 | 131 | 129 | 128 |  | 127 |
|  | ***Dpp4^-/-^*** | Saline | 21 | Pre | 110 | 107 | 105 | 109 | 101 | 109 | 108 | 105 | 101 | 98 | 105 |
|  |  |  |  | Post | 107 | 101 | 90 | 101 | 109 | 102 | 107 | 93 | 109 | 110 | 103 |
|  |  |  | 22 | Pre | 101 | 103 | 107 | 100 | 105 | 111 | 103 | 102 | 99 | 100 | 103 |
|  |  |  |  | Post | 105 | 109 | 103 | 94 | 93 | 124 | 106 | 107 | 94 | 106 | 104 |
|  |  |  | 23 | Pre | 84 | 90 | 82 | 92 | 86 | 89 | 90 | 92 | 95 | 118 | 92 |
|  |  |  |  | Post | 92 | 93 | 92 | 88 | 91 | 91 | 87 | 88 | 77 | 85 | 88 |
|  |  |  | 24 | Pre | 94 | 103 | 93 | 96 | 99 | 93 | 108 | 98 | 91 |  | 97 |
|  |  |  |  | Post | 91 | 96 | 93 | 93 | 91 | 91 | 91 | 98 | 92 |  | 93 |
|  |  |  | 25 | Pre | 115 | 114 | 113 | 110 | 109 | 109 | 106 |  |  |  | 111 |
|  |  |  |  | Post | 114 | 118 | 110 | 116 | 101 | 111 | 105 | 105 | 105 |  | 109 |
|  |  |  | 26 | Pre | 100 | 105 | 97 | 103 | 97 | 93 | 93 | 91 | 98 | 92 | 97 |
|  |  |  |  | Post | ND | | | | | | | | | | |
|  |  |  | 27 | Pre | 88 | 88 | 89 | 90 | 94 | 95 | 97 | 89 | 93 |  | 91 |
|  |  |  |  | Post | 87 | 96 | 90 | 91 | 95 | 89 | 96 | 100 | 99 |  | 94 |
|  |  |  | 28 | Pre | 110 | 112 | 103 | 98 | 110 | 111 | 122 | 105 | 99 |  | 108 |
|  |  |  |  | Post | 101 | 114 | 112 | 111 | 116 | 109 | 101 | 113 | 107 |  | 109 |
|  |  |  | 29 | Pre | 108 | 116 | 113 | 114 | 119 | 115 | 100 | 102 |  |  | 111 |
|  |  |  |  | Post | 118 | 116 | 106 | 109 | 107 | 110 | 100 | 99 | 97 |  | 107 |
|  |  | Ang II | 30 | Pre | 102 | 101 | 105 | 108 | 108 | 103 | 94 | 105 | 105 | 92 | 102 |
|  |  |  |  | Post | ND | | | | | | | | | | |
|  |  |  | 31 | Pre | 98 | 95 | 101 | 95 | 97 | 103 | 105 | 97 | 90 | 92 | 97 |
|  |  |  |  | Post | 107 | 119 | 126 | 106 | 109 | 107 | 108 | 119 | 121 |  | 114 |
|  |  |  | 32 | Pre | 109 | 105 | 101 | 110 | 102 | 109 | 105 | 114 | 105 |  | 107 |
|  |  |  |  | Post | 147 | 148 | 145 | 130 | 127 | 127 | 130 | 147 | 135 | 136 | 137 |
|  |  |  | 33 | Pre | 115 | 110 | 115 | 115 | 112 | 112 | 115 | 111 | 111 | 105 | 112 |
|  |  |  |  | Post | 133 | 133 | 141 | 145 | 124 | 130 | 134 | 134 | 136 | 131 | 134 |
|  |  |  | 34 | Pre | 92 | 98 | 96 | 98 | 96 | 90 | 91 | 85 | 83 | 85 | 91 |
|  |  |  |  | Post | 121 | 118 | 126 | 120 | 117 | 135 | 124 | 125 |  |  | 123 |
|  |  |  | 35 | Pre | 110 | 106 | 104 | 106 | 105 | 105 | 103 | 91 |  |  | 104 |
|  |  |  |  | Post | 135 | 129 | 131 | 131 | 133 | 133 | 134 | 134 | 128 | 125 | 131 |
|  |  |  | 36 | Pre | 93 | 95 | 90 | 93 | 92 | 92 | 98 | 98 |  |  | 94 |
|  |  |  |  | Post | 116 | 115 | 123 | 118 | 121 | 115 | 115 | 129 | 125 |  | 120 |
|  |  |  | 37 | Pre | 92 | 89 | 92 | 89 | 83 | 88 | 79 | 91 |  |  | 88 |
|  |  |  |  | Post | 108 | 106 | 114 | 112 | 116 | 112 | 110 | 112 |  |  | 111 |

ND: not detectable; PA values could not be acquired.

**Supplementary Table S4. Raw systolic blood pressure measurements for CTRL and *Dpp4*^ΔPT^ male mice**

| **MALES** | **Genotype** | **IP injection** | **Mice** |  | **Systolic Blood Pressure Measurements** | | | | | | | | | | **Average** |
| --- | --- | --- | --- | --- | --- | --- | --- | --- | --- | --- | --- | --- | --- | --- | --- |
|  |  |  |  |  | **1** | **2** | **3** | **4** | **5** | **6** | **7** | **8** | **9** | **10** |  |
|  | **CTRL** | Saline | 1 | Pre | 101 | 101 | 110 | 111 | 106 | 102 | 103 | 110 | 110 |  | 106 |
|  |  |  |  | Post | 108 | 103 | 100 | 102 | 110 | 104 | 102 | 110 | 102 |  | 105 |
|  |  |  | 2 | Pre | 100 | 103 | 108 | 110 | 109 | 117 | 101 | 106 | 108 |  | 107 |
|  |  |  |  | Post | 108 | 112 | 102 | 106 | 126 | 116 | 105 | 107 | 111 |  | 110 |
|  |  |  | 3 | Pre | 110 | 109 | 107 | 120 | 109 | 113 | 115 | 109 | 101 |  | 110 |
|  |  |  |  | Post | 107 | 108 | 111 | 109 | 118 | 111 | 105 | 109 | 110 |  | 110 |
|  |  |  | 4 | Pre | 117 | 112 | 126 | 125 | 113 | 109 | 118 | 123 | 120 |  | 118 |
|  |  |  |  | Post | 127 | 123 | 128 | 110 | 106 | 108 | 115 | 112 | 109 |  | 115 |
|  |  |  | 5 | Pre | 119 | 117 | 108 | 115 | 119 | 118 | 120 | 122 | 123 |  | 118 |
|  |  |  |  | Post | 124 | 124 | 125 | 125 | 126 | 113 | 110 | 123 | 119 | 119 | 121 |
|  |  | Ang II | 6 | Pre | 112 | 109 | 110 | 105 | 107 | 113 | 105 | 109 |  |  | 109 |
|  |  |  |  | Post | 134 | 141 | 136 | 134 | 121 | 134 | 133 | 147 | 135 |  | 135 |
|  |  |  | 7 | Pre | 102 | 111 | 113 | 105 | 105 | 112 | 113 | 110 |  |  | 109 |
|  |  |  |  | Post | 154 | 158 | 136 | 130 | 144 | 130 | 128 | 136 | 130 |  | 138 |
|  |  |  | 8 | Pre | 105 | 107 | 106 | 105 | 103 | 106 | 105 | 105 | 103 | 105 | 105 |
|  |  |  |  | Post | 131 | 130 | 129 | 132 | 134 | 132 | 138 | 131 | 134 |  | 132 |
|  |  |  | 9 | Pre | 109 | 107 | 115 | 117 | 113 | 117 | 105 | 103 | 103 | 110 | 110 |
|  |  |  |  | Post | 140 | 137 | 141 | 128 | 135 | 139 | 147 | 134 | 134 | 141 | 138 |
|  |  |  | 10 | Pre | 119 | 115 | 110 | 109 | 108 | 100 | 102 | 110 |  |  | 109 |
|  |  |  |  | Post | 136 | 129 | 139 | 145 | 154 | 138 | 154 | 144 | 142 | 153 | 143 |
|  |  |  | 11 | Pre | 116 | 110 | 114 | 118 | 114 | 119 | 112 | 115 | 105 |  | 114 |
|  |  |  |  | Post | 136 | 142 | 135 | 165 | 137 | 134 | 143 | 141 |  |  | 142 |
|  | ***Dpp4^ΔPT^*** | Saline | 12 | Pre | 117 | 123 | 112 | 102 | 103 | 113 | 109 | 108 |  |  | 111 |
|  |  |  |  | Post | 117 | 106 | 106 | 105 | 109 | 110 | 105 | 107 | 119 |  | 109 |
|  |  |  | 13 | Pre | 114 | 119 | 110 | 112 | 106 | 108 | 109 | 106 |  |  | 111 |
|  |  |  |  | Post | 112 | 109 | 110 | 101 | 115 | 118 | 113 | 111 | 105 |  | 110 |
|  |  |  | 14 | Pre | 108 | 110 | 106 | 107 | 108 | 116 | 121 | 114 |  |  | 111 |
|  |  |  |  | Post | 117 | 109 | 101 | 110 | 115 | 113 | 111 | 103 |  |  | 110 |
|  |  |  | 15 | Pre | 110 | 115 | 109 | 99 | 115 | 123 | 114 | 125 | 100 |  | 112 |
|  |  |  |  | Post | 102 | 110 | 123 | 125 | 123 | 119 | 112 | 109 | 108 |  | 115 |
|  |  |  | 16 | Pre | 114 | 105 | 109 | 108 | 113 | 118 | 114 | 118 | 120 |  | 113 |
|  |  |  |  | Post | 115 | 113 | 114 | 110 | 120 | 121 | 101 | 100 | 109 |  | 111 |
|  |  |  | 17 | Pre | 108 | 113 | 105 | 102 | 99 | 106 | 107 | 128 | 110 | 103 | 108 |
|  |  |  |  | Post | 115 | 103 | 103 | 110 | 108 | 110 | 113 | 115 | 113 |  | 110 |
|  |  | Ang II | 18 | Pre | 108 | 105 | 110 | 111 | 107 | 115 | 115 | 96 | 94 | 99 | 106 |
|  |  |  |  | Post | 134 | 131 | 124 | 125 | 133 | 126 | 122 | 123 |  |  | 127 |
|  |  |  | 19 | Pre | 109 | 112 | 101 | 105 | 102 | 106 | 109 | 108 |  |  | 107 |
|  |  |  |  | Post | 119 | 117 | 125 | 118 | 122 | 122 | 121 | 125 |  |  | 121 |
|  |  |  | 20 | Pre | 104 | 110 | 108 | 112 | 110 | 110 | 100 | 108 | 109 |  | 108 |
|  |  |  |  | Post | 132 | 127 | 128 | 119 | 118 | 122 | 126 | 125 | 124 |  | 125 |
|  |  |  | 21 | Pre | 108 | 110 | 102 | 108 | 106 | 103 | 118 | 118 | 119 | 110 | 110 |
|  |  |  |  | Post | 121 | 118 | 126 | 126 | 123 | 124 | 127 | 123 | 121 | 125 | 123 |
|  |  |  | 22 | Pre | 106 | 115 | 107 | 117 | 109 | 102 | 110 | 102 |  |  | 109 |
|  |  |  |  | Post | 129 | 112 | 133 | 119 | 128 | 128 | 129 | 127 |  |  | 126 |

**Supplementary Table S5. Raw systolic blood pressure measurements for CTRL and *Dpp4*^ΔPT^ female mice**

| **FEMALES** | **Genotype** | **IP injection** | **Mice** | **Measurement** | **Systolic Blood Pressure Measurements** | | | | | | | | | | **Average** |
| --- | --- | --- | --- | --- | --- | --- | --- | --- | --- | --- | --- | --- | --- | --- | --- |
|  |  |  |  |  | **1** | **2** | **3** | **4** | **5** | **6** | **7** | **8** | **9** | **10** |  |
|  | **CTRL** | Saline | 1 | Pre | 95 | 98 | 105 | 99 | 105 | 110 | 98 | 110 | 105 |  | 103 |
|  |  |  |  | Post | 103 | 105 | 105 | 103 | 101 | 106 | 107 | 107 | 104 | 107 | 105 |
|  |  |  | 2 | Pre | 99 | 103 | 108 | 101 | 96 | 99 | 93 | 100 | 101 |  | 100 |
|  |  |  |  | Post | 101 | 105 | 101 | 108 | 107 | 106 | 103 | 104 | 102 |  | 104 |
|  |  |  | 3 | Pre | 94 | 101 | 98 | 93 | 98 | 102 | 105 | 101 | 98 | 99 | 99 |
|  |  |  |  | Post | 97 | 110 | 105 | 102 | 101 | 98 | 99 | 102 | 101 | 103 | 102 |
|  |  |  | 4 | Pre | 89 | 94 | 89 | 91 | 93 | 92 | 98 | 101 | 102 | 93 | 94 |
|  |  |  |  | Post | 101 | 93 | 99 | 100 | 96 | 98 | 96 | 101 | 102 |  | 98 |
|  |  |  | 5 | Pre | 99 | 104 | 101 | 99 | 89 | 94 | 102 | 100 | 99 | 98 | 99 |
|  |  |  |  | Post | 100 | 89 | 92 | 101 | 95 | 98 | 97 | 101 | 103 |  | 97 |
|  |  | Ang II | 12 | Pre | 101 | 105 | 97 | 97 | 104 | 96 | 101 | 103 |  |  | 101 |
|  |  |  |  | Post | 137 | 128 | 133 | 133 | 131 | 123 | 137 | 127 | 131 | 133 | 131 |
|  |  |  | 13 | Pre | 91 | 85 | 95 | 90 | 95 | 94 | 99 | 107 | 97 | 101 | 95 |
|  |  |  |  | Post | 114 | 116 | 115 | 118 | 114 | 116 | 114 | 118 | 114 | 113 | 115 |
|  |  |  | 14 | Pre | 95 | 92 | 95 | 97 | 93 | 103 | 101 | 102 | 96 | 95 | 97 |
|  |  |  |  | Post | 122 | 122 | 124 | 126 | 129 | 123 | 128 | 127 |  |  | 125 |
|  |  |  | 15 | Pre | 82 | 88 | 96 | 78 | 85 | 88 | 97 | 89 | 86 |  | 88 |
|  |  |  |  | Post | 125 | 121 | 117 | 123 | 121 | 129 | 124 | 127 | 118 | 119 | 122 |
|  |  |  | 16 | Pre | 102 | 98 | 96 | 98 | 100 | 93 | 101 | 103 | 96 | 95 | 98 |
|  |  |  |  | Post | 133 | 132 | 133 | 131 | 127 | 126 | 128 | 125 | 127 | 129 | 129 |
|  |  |  | 17 | Pre | 112 | 105 | 108 | 109 | 110 | 106 | 101 | 100 | 102 |  | 106 |
|  |  |  |  | Post | 133 | 129 | 127 | 130 | 135 | 129 | 130 | 131 | 136 |  | 131 |
|  | ***Dpp4^ΔPT^*** | Saline | 18 | Pre | 109 | 101 | 103 | 105 | 106 | 103 | 110 | 106 |  |  | 105 |
|  |  |  |  | Post | 105 | 108 | 109 | 106 | 108 | 105 | 110 |  |  |  | 107 |
|  |  |  | 19 | Pre | 105 | 101 | 102 | 113 | 105 | 110 | 109 | 101 |  |  | 106 |
|  |  |  |  | Post | 98 | 101 | 99 | 99 | 108 | 109 | 101 | 110 | 115 |  | 104 |
|  |  |  | 20 | Pre | 88 | 85 | 85 | 86 | 96 | 96 | 97 | 92 | 98 | 92 | 92 |
|  |  |  |  | Post | 83 | 89 | 89 | 96 | 98 | 98 | 98 | 96 |  |  | 93 |
|  |  |  | 21 | Pre | 96 | 107 | 101 | 99 | 101 | 99 | 98 | 99 | 99 |  | 100 |
|  |  |  |  | Post | 92 | 93 | 105 | 108 | 107 | 101 | 102 | 99 | 106 |  | 101 |
|  |  |  | 22 | Pre | 108 | 99 | 105 | 105 | 103 | 94 | 92 | 96 | 95 | 91 | 99 |
|  |  |  |  | Post | 99 | 100 | 96 | 95 | 96 | 93 | 91 | 92 | 96 | 98 | 96 |
|  |  | Ang II | 23 | Pre | 97 | 82 | 98 | 83 | 95 | 94 | 92 | 96 |  |  | 92 |
|  |  |  |  | Post | 103 | 118 | 106 | 113 | 106 | 109 | 110 | 109 |  |  | 109 |
|  |  |  | 24 | Pre | 101 | 95 | 102 | 110 | 109 | 110 | 109 | 103 | 105 | 106 | 105 |
|  |  |  |  | Post | 126 | 124 | 128 | 129 | 124 | 126 | 125 | 122 | 122 | 119 | 125 |
|  |  |  | 25 | Pre | 99 | 93 | 101 | 108 | 106 | 109 | 110 | 108 | 100 | 110 | 104 |
|  |  |  |  | Post | 126 | 127 | 136 | 131 | 129 | 124 | 131 | 129 | 123 |  | 128 |
|  |  |  | 26 | Pre | 98 | 118 | 102 | 106 | 106 | 100 | 96 | 106 | 99 | 101 | 103 |
|  |  |  |  | Post | 117 | 117 | 129 | 113 | 126 | 129 | 127 | 121 | 115 | 118 | 121 |
|  |  |  | 27 | Pre | 111 | 108 | 104 | 105 | 102 | 105 | 107 | 102 | 107 |  | 106 |
|  |  |  |  | Post | 122 | 130 | 131 | 124 | 121 | 124 | 124 | 129 |  |  | 126 |

**Supplementary Table S6.** Body weight, volume of injected saline solution, and raw measurements of Figure 2C, E.

| **Genotype** | **Sex** | **Mice** | **Body weight (g)** | **Volume injected of NaCl 0.9% (ml)** | **Volume excreted (μl)** | **Volume excreted (ul/g body weight)** | **Urinary volume (% injected)** | **Unine Na^+^ (mEq/l)** | **CENa^+^ excreted (mEq/g body weight)** | **Urinary Na^+^ (% injected)** |
| --- | --- | --- | --- | --- | --- | --- | --- | --- | --- | --- |
| **CTRL** | Male | 1 | 27.48 | 2.75 | 816 | 29.69 | 30.22 | 134 | 4.88 | 26.30 |
|  | Male | 2 | 31.85 | 3.19 | 1055 | 33.12 | 32.97 | 137 | 4.30 | 29.33 |
|  | Male | 3 | 32.60 | 3.26 | 1055 | 32.36 | 31.97 | 168 | 5.15 | 34.88 |
|  | Male | 4 | 31.53 | 3.15 | 694 | 22.01 | 21.69 | 163 | 5.17 | 22.95 |
|  | Male | 5 | 25.90 | 2.59 | 900 | 34.75 | 34.62 | 152 | 5.87 | 34.17 |
|  | Male | 6 | 27.57 | 2.76 | 589 | 21.36 | 21.04 | 170 | 6.17 | 23.22 |
|  | Male | 7 | 26.04 | 2.60 | 762 | 29.26 | 29.31 | 157 | 6.03 | 29.88 |
|  | Male | 8 | 29.43 | 2.94 | 832 | 28.27 | 28.27 | NA | NA | NA |
| ***Dpp4^ΔPT^*** | Male | 9 | 30.98 | 3.10 | 1355 | 43.74 | 43.71 | 154 | 4.97 | 43.71 |
|  | Male | 10 | 31.40 | 3.14 | 1717 | 54.68 | 55.39 | 153 | 4.87 | 55.03 |
|  | Male | 11 | 28.36 | 2.84 | 1503 | 53.00 | 53.68 | 148 | 5.22 | 51.59 |
|  | Male | 12 | 23.25 | 2.33 | 1017 | 43.74 | 44.22 | 208 | 8.95 | 59.72 |
|  | Male | 13 | 24.80 | 2.48 | 1824 | 73.55 | 72.96 | 155 | 6.25 | 73.43 |
|  | Male | 14 | 25.72 | 2.57 | 2177.2 | 84.65 | 83.74 | 156 | 6.07 | 84.83 |
|  | Male | 15 | 29.14 | 2.91 | 1360 | 46.67 | 46.90 | 221 | 7.58 | 67.30 |
|  | Male | 16 | 24.49 | 2.45 | 1854 | 75.70 | 74.16 | 161 | 6.57 | 77.53 |
| **CTRL** | Female | 1 | 21.48 | 2.15 | 716 | 33.33 | 32.55 | 166 | 7.73 | 35.08 |
|  | Female | 2 | 22.65 | 2.27 | 853 | 37.66 | 37.09 | 155 | 6.84 | 37.33 |
|  | Female | 3 | 21.01 | 2.10 | 743 | 35.36 | 35.38 | 195 | 9.28 | 44.80 |
|  | Female | 4 | 19.83 | 1.98 | 886 | 44.68 | 44.30 | 190 | 9.58 | 54.66 |
|  | Female | 5 | 21.91 | 2.19 | 1000 | 45.64 | 45.45 | 163 | 7.44 | 48.11 |
|  | Female | 6 | 21.95 | 2.20 | 845 | 38.50 | 38.41 | 213 | 9.70 | 53.12 |
|  | Female | 7 | 20.40 | 2.04 | 757 | 37.11 | 37.85 | 174 | 8.53 | 42.77 |
|  | Female | 8 | 21.82 | 2.18 | 1055 | 48.35 | 47.95 | 170 | 7.79 | 52.94 |
|  | Female | 9 | 21.15 | 2.12 | 684 | 32.34 | 32.57 | 189 | 8.94 | 39.97 |
| ***Dpp4^ΔPT^*** | Female | 10 | 24.61 | 2.46 | 1055 | 42.87 | 42.20 | 142 | 5.77 | 38.91 |
|  | Female | 11 | 22.89 | 2.29 | 1045 | 45.65 | 45.43 | 170 | 7.43 | 50.16 |
|  | Female | 12 | 21.24 | 2.12 | 1182 | 55.65 | 56.29 | 205 | 9.65 | 74.93 |
|  | Female | 13 | 20.53 | 2.05 | 1075 | 52.36 | 51.19 | 174 | 8.48 | 57.84 |
|  | Female | 14 | 20.85 | 2.09 | 1086 | 52.09 | 51.71 | 191 | 9.16 | 64.14 |
|  | Female | 15 | 21.49 | 2.15 | 989 | 46.02 | 44.95 | 184 | 8.56 | 53.71 |
|  | Female | 16 | 21.96 | 2.20 | 1255 | 57.15 | 57.05 | 217 | 9.88 | 80.38 |
|  | Female | 17 | 20.87 | 2.09 | 976 | 46.77 | 46.48 | 184 | 8.82 | 55.53 |
|  | Female | 18 | 21.81 | 2.18 | 1561 | 71.57 | 70.95 | 184 | 8.44 | 84.78 |

NA: Not available. The samples failed to meet the manufacturer's quality standards for the technique, preventing the analysis from being performed.

**Supplementary Table S7.** Body weight, volume of injected saline solution, and raw measurements of Figure 2D, F.

| **Genotype** | **Sex** | **Mice** | **Body weight (g)** | **Volume injected of NaCl 0.9% (ml)** | **Volume excreted (μl)** | **Volume excreted (μl/g body weight)** | **Urinary volume (% injected)** | **Unine Na^+^ (mEq/l)** | **CENa^+^ excreted (mEq/g body weight)** | **Urinary Na^+^ (% injected)** |
| --- | --- | --- | --- | --- | --- | --- | --- | --- | --- | --- |
| **WT** | Male | 1 | 31.78 | 3.18 | 761 | 23.95 | 23.95 | 128 | 4.03 | 19.90 |
|  | Male | 2 | 31.11 | 3.11 | 757 | 24.33 | 24.33 | 128 | 4.11 | 20.22 |
|  | Male | 3 | 28.64 | 2.86 | 892 | 31.15 | 31.15 | 141 | 4.92 | 28.52 |
|  | Male | 4 | 27.08 | 2.71 | 769 | 28.40 | 28.40 | 139 | 5.13 | 25.63 |
|  | Male | 5 | 28.23 | 2.82 | 905 | 32.06 | 32.06 | 134 | 4.75 | 27.89 |
|  | Male | 6 | 29.43 | 2.94 | 832 | 28.27 | 28.27 | 134 | 4.55 | 24.60 |
|  | Male | 7 | 33.13 | 3.31 | 642 | 19.38 | 19.38 | 142 | 4.29 | 17.87 |
|  | Male | 8 | 28.85 | 2.89 | 864 | 29.95 | 29.95 | 105 | 3.64 | 20.42 |
|  | Male | 9 | 29.75 | 2.98 | 964 | 32.40 | 32.40 | 135 | 4.54 | 28.41 |
| ***Dpp4^-/-^*** | Male | 10 | 26.81 | 2.68 | 1422 | 53.04 | 53.04 | 120 | 4.48 | 46.04 |
|  | Male | 11 | 30.77 | 3.08 | 1818 | 59.08 | 59.08 | 142 | 4.61 | 39.77 |
|  | Male | 12 | 28.96 | 2.90 | 1249 | 43.13 | 43.13 | 138 | 4.77 | 53.08 |
|  | Male | 13 | 27.45 | 2.75 | 1626 | 59.23 | 59.23 | 122 | 4.44 | 33.81 |
|  | Male | 14 | 30.93 | 3.09 | 1320 | 42.68 | 42.68 | 218 | 7.05 | 73.09 |
|  | Male | 15 | 26.90 | 2.69 | 1389 | 51.64 | 51.64 | 184 | 6.84 | 61.42 |
|  | Male | 16 | 27.66 | 2.77 | 1422 | 51.41 | 51.41 | 146 | 5.28 | 49.71 |
|  | Male | 17 | 29.83 | 2.98 | 1564 | 52.43 | 52.43 | NA | NA | NA |
|  | Male | 18 | 30.10 | 3.01 | 1506 | 50.03 | 50.03 | NA | NA | NA |
| **WT** | Female | 1 | 21.64 | 2.16 | 704 | 32.53 | 32.53 | 138 | 6.38 | 29.15 |
|  | Female | 2 | 22.46 | 2.25 | 734 | 32.68 | 32.68 | 175 | 7.79 | 37.14 |
|  | Female | 3 | 24.61 | 2.46 | 741 | 30.11 | 30.11 | 149 | 6.05 | 29.13 |
|  | Female | 4 | 22.13 | 2.21 | 780 | 35.25 | 35.25 | 122 | 5.51 | 27.92 |
|  | Female | 5 | 24.70 | 2.47 | 920 | 37.25 | 37.25 | 167 | 6.76 | 40.39 |
|  | Female | 6 | 18.16 | 1.82 | 848 | 46.70 | 46.70 | 160 | 8.81 | 48.52 |
|  | Female | 7 | 24.97 | 2.50 | 998 | 39.97 | 39.97 | 156 | 6.25 | 40.49 |
|  | Female | 8 | 23,54 | 2,35 | 960 | 40,78 | 40,78 | 142 | 6,03 | 37,60 |
| ***Dpp4^-/-^*** | Female | 9 | 20,41 | 2,04 | 893 | 43,75 | 43,75 | 180 | 8,82 | 50,71 |
|  | Female | 10 | 22.15 | 2.22 | 961 | 43.39 | 43.39 | 153 | 6.91 | 41.33 |
|  | Female | 11 | 22.01 | 2.20 | 1088 | 49.43 | 49.43 | 179 | 6.95 | 67.76 |
|  | Female | 12 | 23.70 | 2.37 | 1004 | 42.36 | 42.36 | 131 | 7.55 | 50.48 |
|  | Female | 13 | 21.08 | 2.11 | 877 | 41.60 | 41.60 | 118 | 6.21 | 48.44 |
|  | Female | 14 | 22.06 | 2.21 | 1286 | 58.30 | 58.30 | 165 | 5.35 | 55.14 |
|  | Female | 15 | 21.40 | 2.14 | 1270 | 59.35 | 59.35 | 162 | 7.71 | 57.83 |
|  | Female | 16 | 22.62 | 2.26 | 1430 | 63.22 | 63.22 | NA | NA | NA |
|  | Female | 17 | 23.26 | 2.33 | 1197 | 51.46 | 51.46 | NA | NA | NA |
|  | Female | 18 | 21.50 | 2.15 | 1182 | 54.98 | 54.98 | NA | NA | NA |

NA: Not available. The samples failed to meet the manufacturer's quality standards for the technique, preventing the analysis from being performed.

**Supplementary Table S8.** Antibody details. IB: immunoblotting, IF: immunofluorescence.

| **Target** | **Method** | **Species raised** | **Molecular weight (kDa)** | **μg protein/**  **lane** | **1^ARY^antibody  specifications** | **2^ARY^ antibody  specifications** |
| --- | --- | --- | --- | --- | --- | --- |
| **DPP4** | IB | Goat | 110 | 10 | AF954  R&D Systems (Minneapolis, MN) 1:800 v/v | 305-035-003  Jackson Immuno Research  Peroxidase AffiniPure™ (HRP) Rabbit Anti-Goat IgG 1:2000 v/v |
| **NHE3** | IB | Rabbit | 80 | 20 | Alicia McDonough (USC, Los Angeles, CA) 1:2000 v/v | 111-035-144  Jackson Immuno Research  Peroxidase AffiniPure™ (HRP) Goat Anti-Rabbit IgG  1:2000 v/v |
| **pS552-NHE3** | IB | Mouse | 80 | 10 | Sc-53962 Santa Cruz Biotechnology  (Santa Cruz, CA) 1:1000 v/v | 115-035-062  Jackson Immuno Research  Peroxidase AffiniPure™ (HRP) Goat Anti-Mouse IgG  1:2000 v/v |
| **NCC** | IB | Rabbit | 130-150 | 20 | Alicia McDonough (USC, Los Angeles, CA) 1:2000 v/v | 111-035-144  Jackson Immuno Research  Peroxidase AffiniPure™ (HRP) Goat Anti-Rabbit IgG  1:2000 v/v |
| **pT53-NCC** | IB | Rabbit | 160 | 20 | SKU: p1311-53  Phosphosolutions  (Aurora, CO) 1:5000 v/v | 111-035-144  Jackson Immuno Research  Peroxidase AffiniPure™ (HRP) Goat Anti-Rabbit IgG  1:2000 v/v |
| **α - ENaC** | IB | Rabbit | 85/30 | 20 | SPC-403  StressMarq Biosciences (British Columbia, Canada)  1:1000 v/v | 111-035-144  Jackson Immuno Research  Peroxidase AffiniPure™ (HRP) Goat Anti-Rabbit IgG  1:2000 v/v |
| **γ - ENaC** | IB | Rabbit | 80/60 | 20 | SPC-405  StressMarq Biosciences (British Columbia, Canada)  1:1000 v/v | 111-035-144  Jackson Immuno Research  Peroxidase AffiniPure™ (HRP) Goat Anti-Rabbit IgG  1:2000 v/v |
| **DPP4** | IF | Goat | - | - | AF954  R&D Systems (Minneapolis, MN) 1:100 v/v | A11055  Life Technologies  Alexa Fluor^TM^ 488 Donkey anti-goat IgG  1:500 v/v |
| **SGLT2** | IF | Rabbit | - | - | 20802  Bicell  (St. Louis, MO)  1:100 v/v | A31573  Life Technologies  Alexa Fluor^TM^ 647 Donkey anti-rabbit IgG  1:500 v/v |

**Supplementary Table S9.** Power analysis for primary and secondary outcomes^1^.

| **Parameter** | **Effect size (f)** | **α** | **Sample size used (per group)** | **Post hoc power (1-β)** | **Interpretation** |
| --- | --- | --- | --- | --- | --- |
| **Baseline analysis** | | | | |  |
| SBP | 1.26 | 0.05 | 10–11 | ~1.00 | Very large |
| Diuresis | 1.39 | 0.05 | 8–12 | ~1.00 | Very large |
| Natriuresis | 1.39 | 0.05 | 7–9 | ~1.00 | Very large |
| pS552/NHE3 ratio | 2.85 | 0.05 | 5–6 | ~1.00 | Extremely large |
| **Males - post Ang II injection analysis** | | | | |  |
| Delta SBP | 5.83 | 0.05 | 5–6 | ~1.00 | Extremely large |
| Renal DPP4 activity | 7.67 | 0.05 | 5–6 | ~1.00 | Extremely large |
| pS552/NHE3 ratio | 2.51 | 0.05 | 5–6 | ~1.00 | Extremely large |
| **Females - post Ang II injection analysis** | | | | |  |
| Delta SBP | 4.55 | 0.05 | 5–6 | ~1.00 | Extremely large |
| Renal DPP4 activity | 4.71 | 0.05 | 5–6 | ~1.00 | Extremely large |
| pS552/NHE3 ratio | 2.50 | 0.05 | 5–6 | ~1.00 | Extremely large |

^1^Faul, F., Erdfelder, E., Lang, A.-G., & Buchner, A. (2007). G*Power 3: A flexible statistical power analysis program for the social, behavioral, and biomedical sciences. Behavior Research Methods, 39, 175-191.


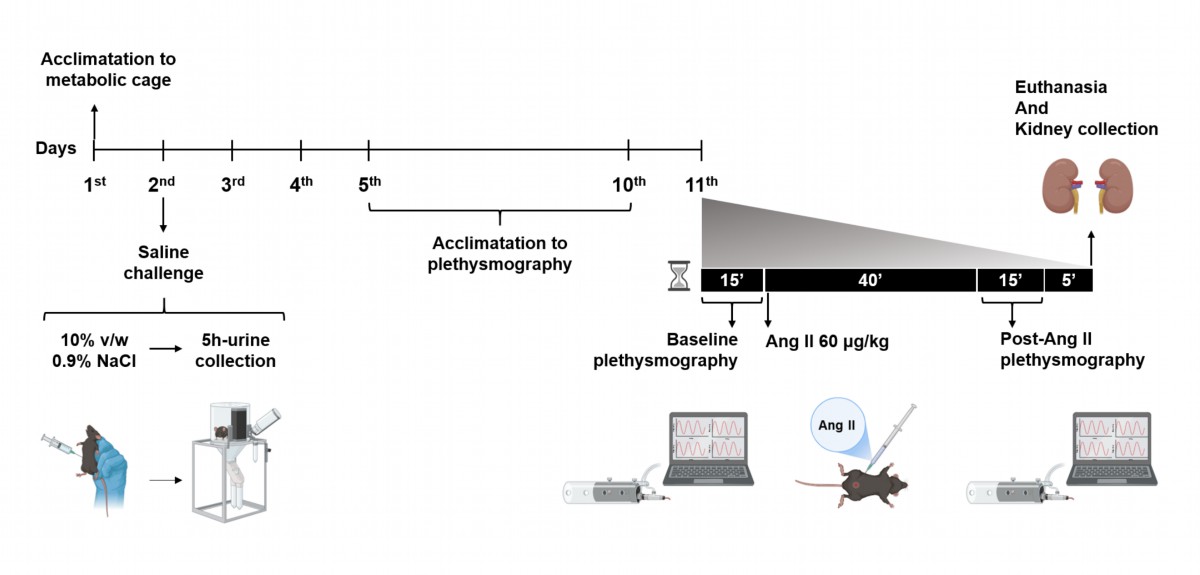


**Supplementary Figure S1. Experimental design –** Experiments were conducted in 12-week-old male and female mice. Following a 24-hour acclimation period in metabolic cages, mice received an intraperitoneal injection of 0.9% NaCl. The next two days served as a rest period with no mouse manipulation. Afterward, mice were acclimated for noninvasive systolic blood pressure (BP) measurement via tail-cuff plethysmography over six consecutive mornings, each lasting approximately 10 minutes. On the sixth day, SBP was measured and designated as the baseline. Subsequently, mice received an intraperitoneal infusion of Ang II (60 ng/kg) or saline, were returned to their cages for 40 minutes, and underwent a 15-minute SBP measurement session via tail-cuff plethysmography. Kidneys were collected in ice-cold PBS containing protease and phosphatase inhibitors or fixed in 4% paraformaldehyde for further analysis.


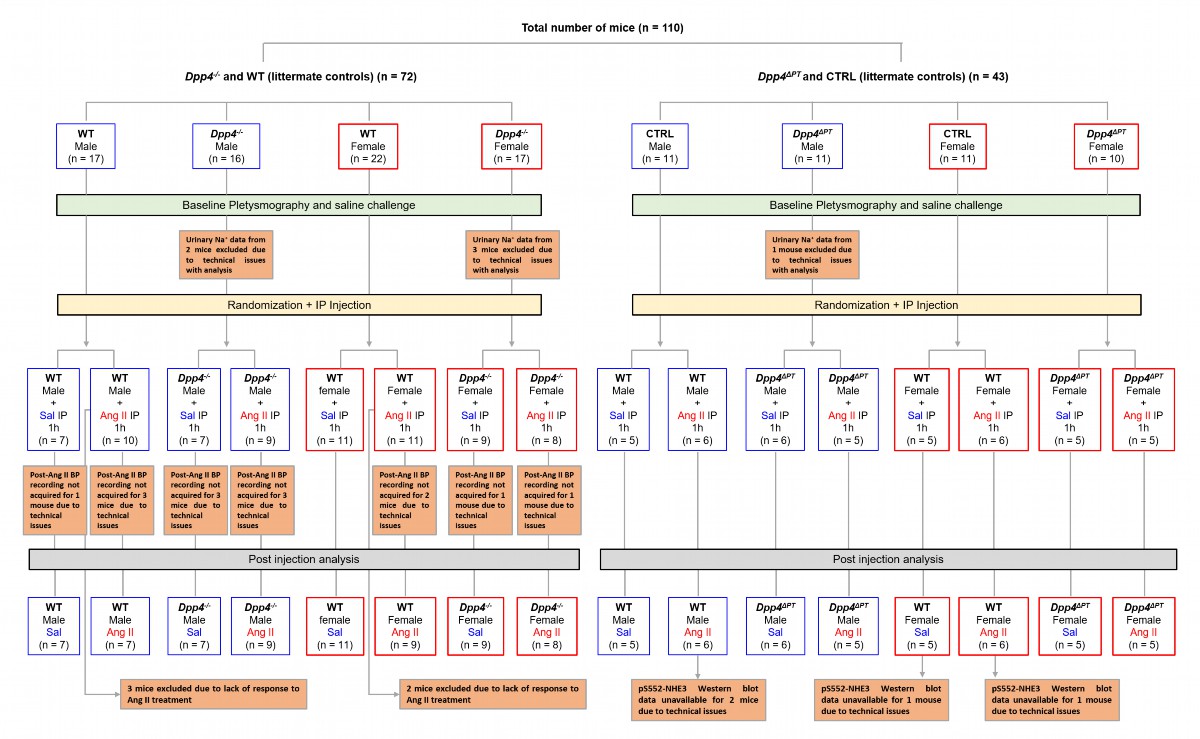


**Supplementary Figure S2. CONSORT-style diagram.**


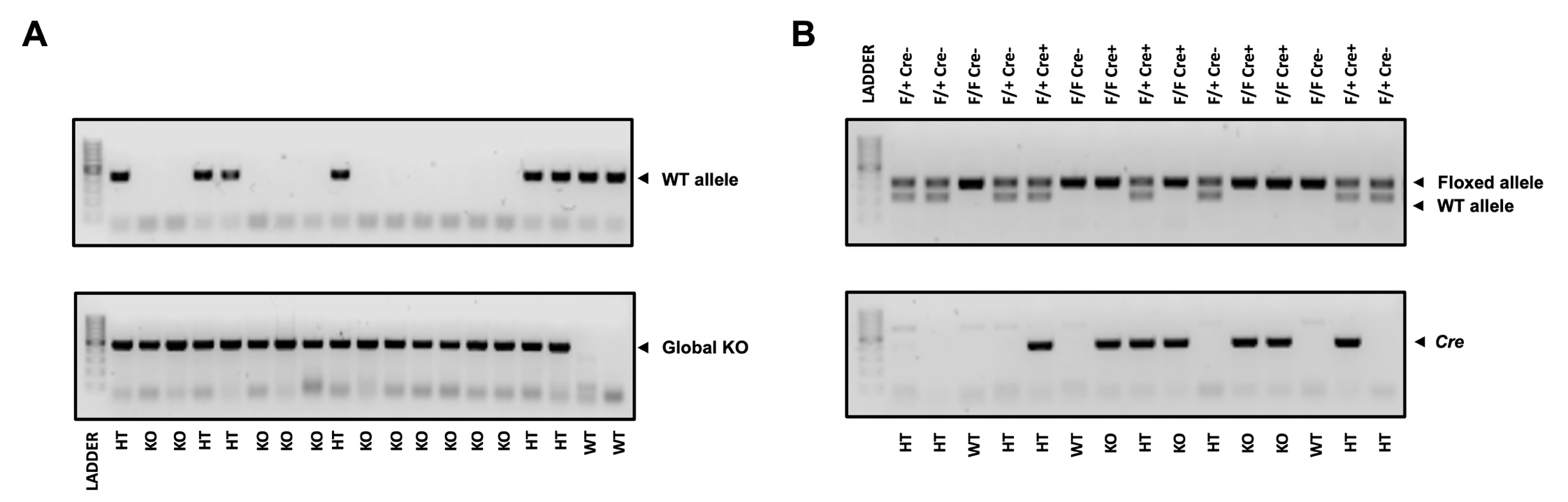


**Supplementary Figure S3 – Genotyping of PT-Specific and global *Dpp4* knockout mice.** **(A)** Genotyping of global *Dpp4* knockout (*Dpp4^-/-^*) mice. The top PCR reaction detects the *Dpp4* wild-type allele, and the bottom reaction was performed to detect the mutated *Dpp4* allele. Heterozygous (HT) mice show products in both reactions, while wild-type (WT) mice only show a product in the top reaction. Global *Dpp4* knockout mice (KO) show a product only in the bottom reaction and are referred to as *Dpp4*^-/-^ throughout the manuscript. **(B)**Genotyping of PT-specific *Dpp4*knockout (*Dpp4*^ΔPT^) mice. The top PCR reaction detects the wild type and floxed (F) *Dpp4*alleles, while the bottom reaction detects the presence of *Cre*. F/F; *Cre*^—^ mice are wild type littermates (WT), represented throughout the manuscript as "CTRL"; F/F; *Cre*^+^ mice are conditional knockouts (KO), referred to as *Dpp4*^ΔPT^. F/+; *Cre*^+^ were heterozygous (HT) mice, while F/+; *Cre*^-^ were also wild type (WT).


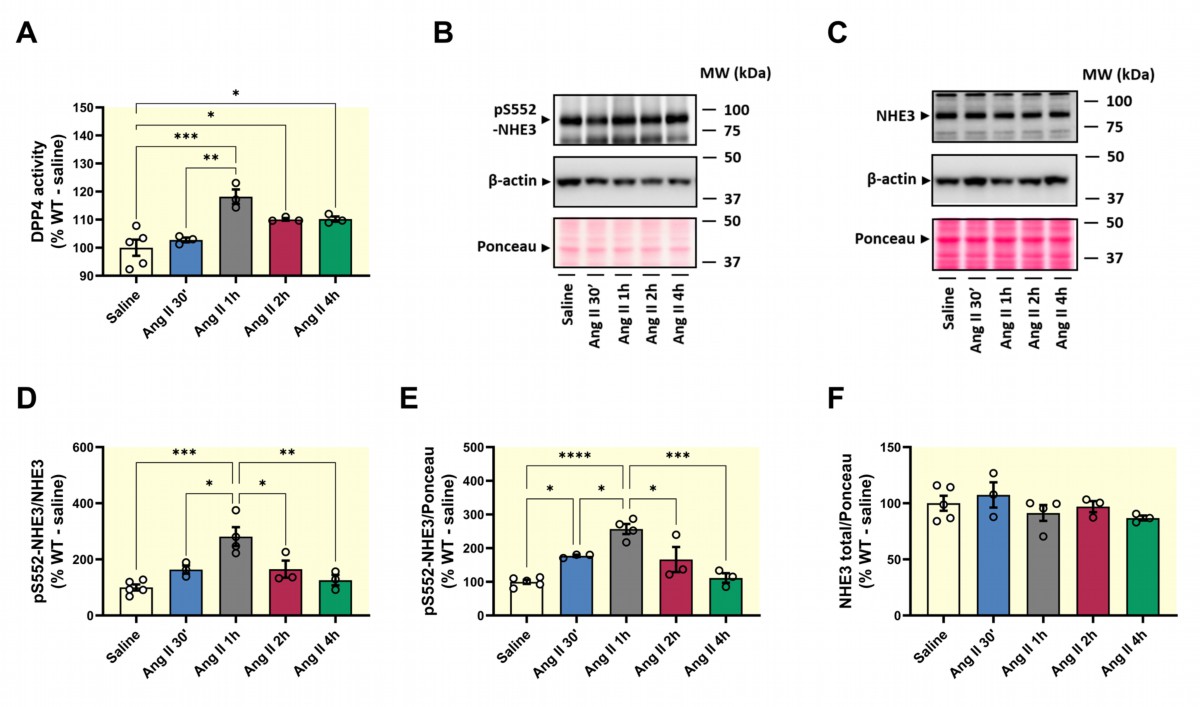


**Supplementary Figure S4 – Standardization of the experimental protocol for acute Ang II administration.** A pressor dose of Ang II (1000 ng/kg/min, equivalent to 60 µg/kg) was administered intraperitoneally to male WT mice. **(A)** DPP4 activity measured by fluorimetry. **(B)** Immunoblot evaluation of pS552-NHE3. **(C)**Immunoblot evaluation of total NHE3 expression. **(D)** pS552-NHE3/NHE3 ratio. **(E)** Quantification of pS552-NHE3 normalized by Ponceau staining. **(F)** Quantification of total NHE3 normalized by Ponceau staining. Values are expressed as a percentage of WT and presented as mean ± SEM. Data were tested for normality using the Shapiro-Wilk test. The experimental n ranged from 3 to 5. One-way ANOVA followed by Tukey's post-test was used for comparisons. *P < 0.05, **P < 0.01, ***P < 0.001 and ****P < 0.0001.


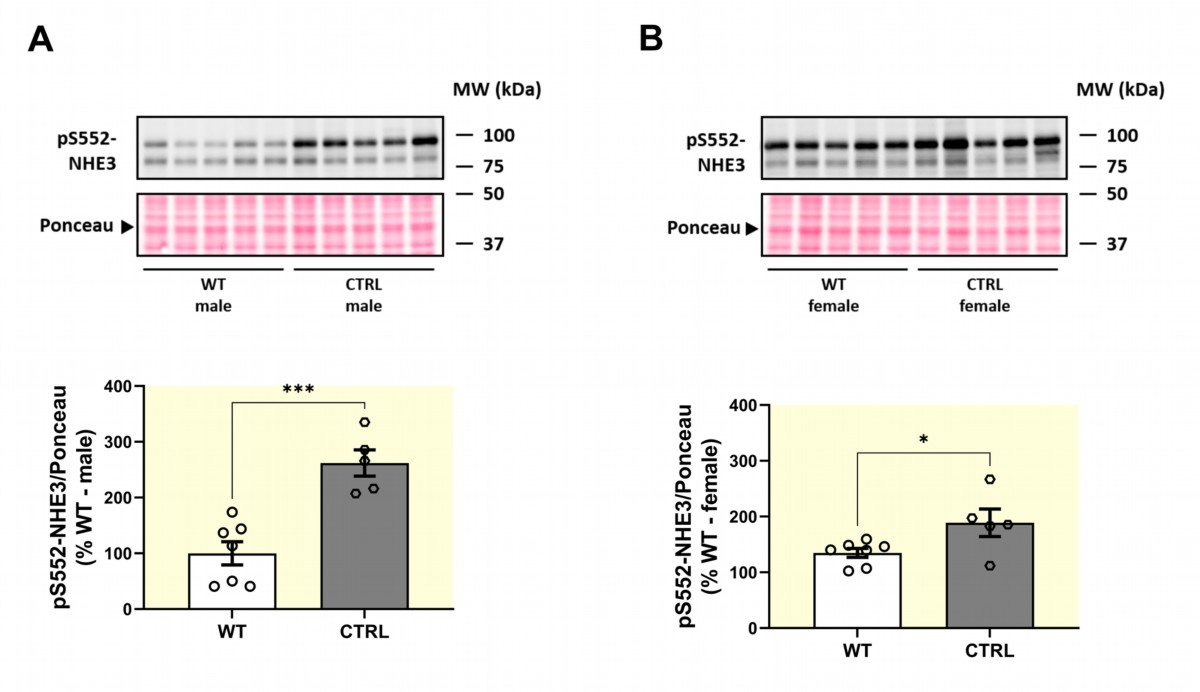


**Supplementary Figure S5 – Baseline differences in NHE3 phosphorylation at serine 552 in male and female CTRL and WT mice. Evaluation of the levels of** NHE3 phosphorylation at serine 552 in kidney homogenates from **(A)** male or **(B)** female CTRL (*Dpp4*^ΔPT^ littermate) and WT (*Dpp4*^-/-^ littermate) mice. Densitometric analyses were normalized to the Ponceau signal (~42 kDa). Data are expressed as a percentage of the respective WT (male or female) controls and shown as mean ± SEM. Values were tested for normality using the Shapiro-Wilk test. The experimental n ranged from 5 to 7. Statistical significance was determined by the Student's t-test. *P < 0.05 and ***P < 0.001.


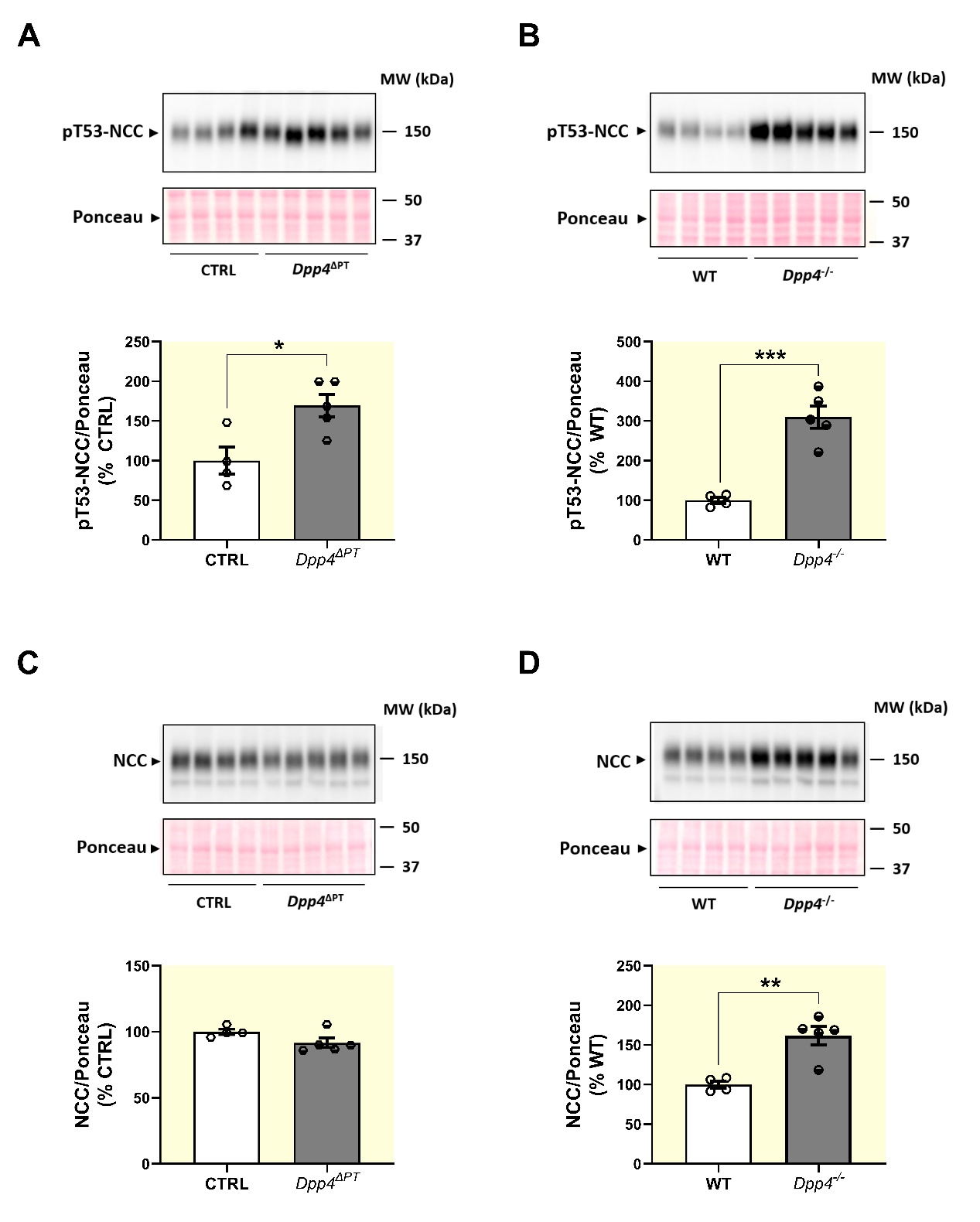


**Supplementary Figure S6 – Total NCC abundance and NCC phosphorylation at threonine 53 (pT53-NCC) in male *Dpp4*^ΔPT^ and *Dpp4*^-/-^ mice.** Total NCC abundance and phosphorylation at T53 were analyzed by immunoblotting in kidney homogenates from male mice. **(A)** Phosphorylation levels of NCC at threonine 53 in CTRL and *Dpp4^ΔPT^* mice. **(B)** Phosphorylation levels of NCC at threonine 53 in WT and *Dpp4*^-/-^ mice. **(C)** Total NCC abundance of CTRL and *Dpp4*^ΔPT^ mice. **(D)** Total NCC abundance of WT and *Dpp4*^-/-^ mice. Densitometry results were normalized to Ponceau staining (~42 kDa). Data expressed as mean ± SEM, with dots representing the % of CTRL or WT males for each animal. Data were tested for normality using the Shapiro-Wilk test. The experimental n ranged from 4 to 5. Statistical analysis was performed using Student's t-test. *P < 0.05, **P < 0.01 and ***P < 0.001.


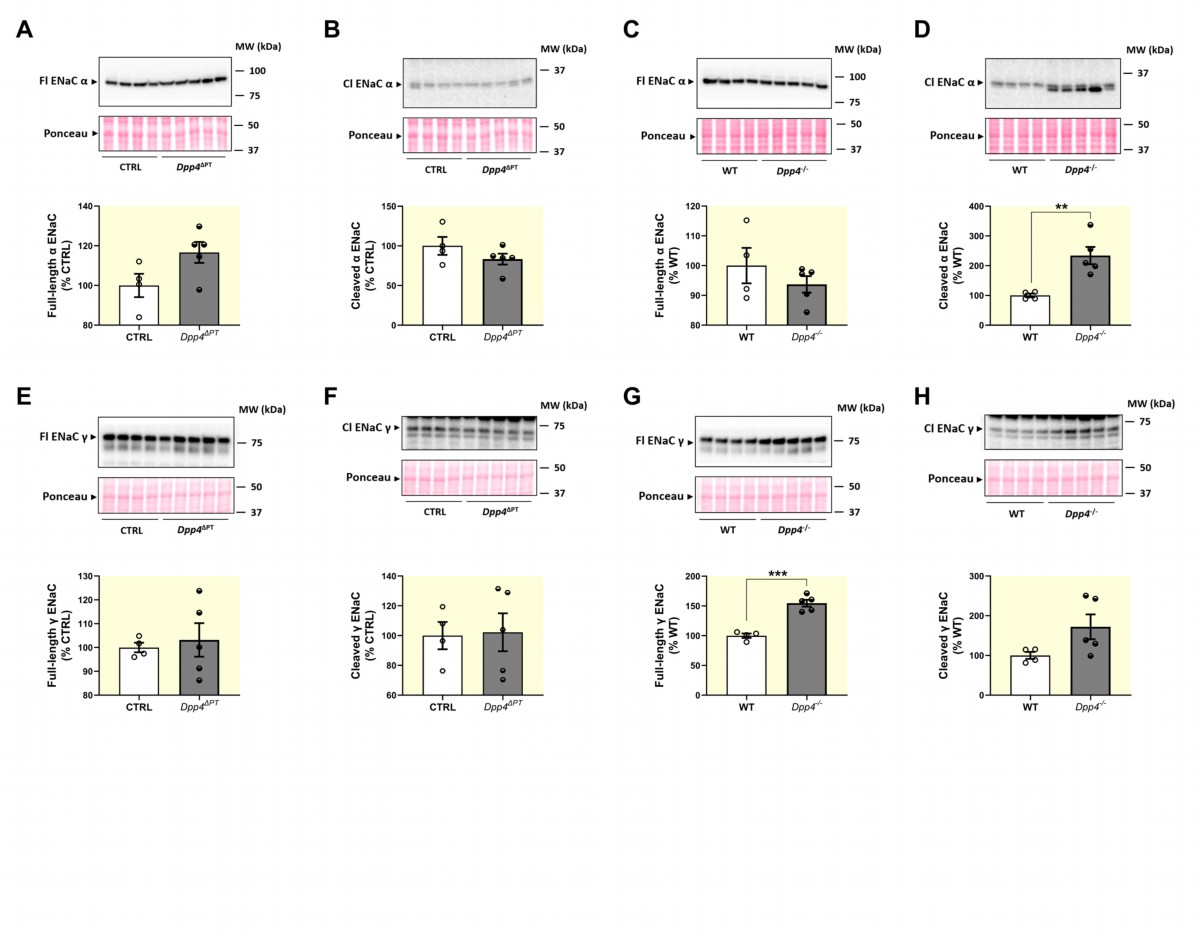


**Supplementary Figure S7 – Abundance of full-length and cleaved ENaC subunits α and γ in kidneys from male *Dpp4^ΔPT^* and *Dpp4^-/-^* mice.** Representative immunoblots and relative expression of **(A)** full-length α ENaC and **(B)** cleaved α ENaC from *Dpp4^ΔPT^* mice, **(C)** full-length α ENaC and **(D)** cleaved α ENaC from *Dpp4^-/-^* mice, **(E)** full-length γ ENaC and **(F)** cleaved γ ENaC from *Dpp4^ΔPT^* mice, **(G)** full-length γ ENaC and **(H)** cleaved γ ENaC from *Dpp4^-/-^* mice. Densitometry results were normalized to Ponceau staining (~42 kDa). Data are expressed as mean ± SEM, with dots representing the % of CTRL or WT for each animal. Data were tested for normality using the Shapiro-Wilk test. The experimental n ranged from 4 to 5. Statistical analysis was performed using Student's t-test. **P < 0.01, ***P < 0.001.


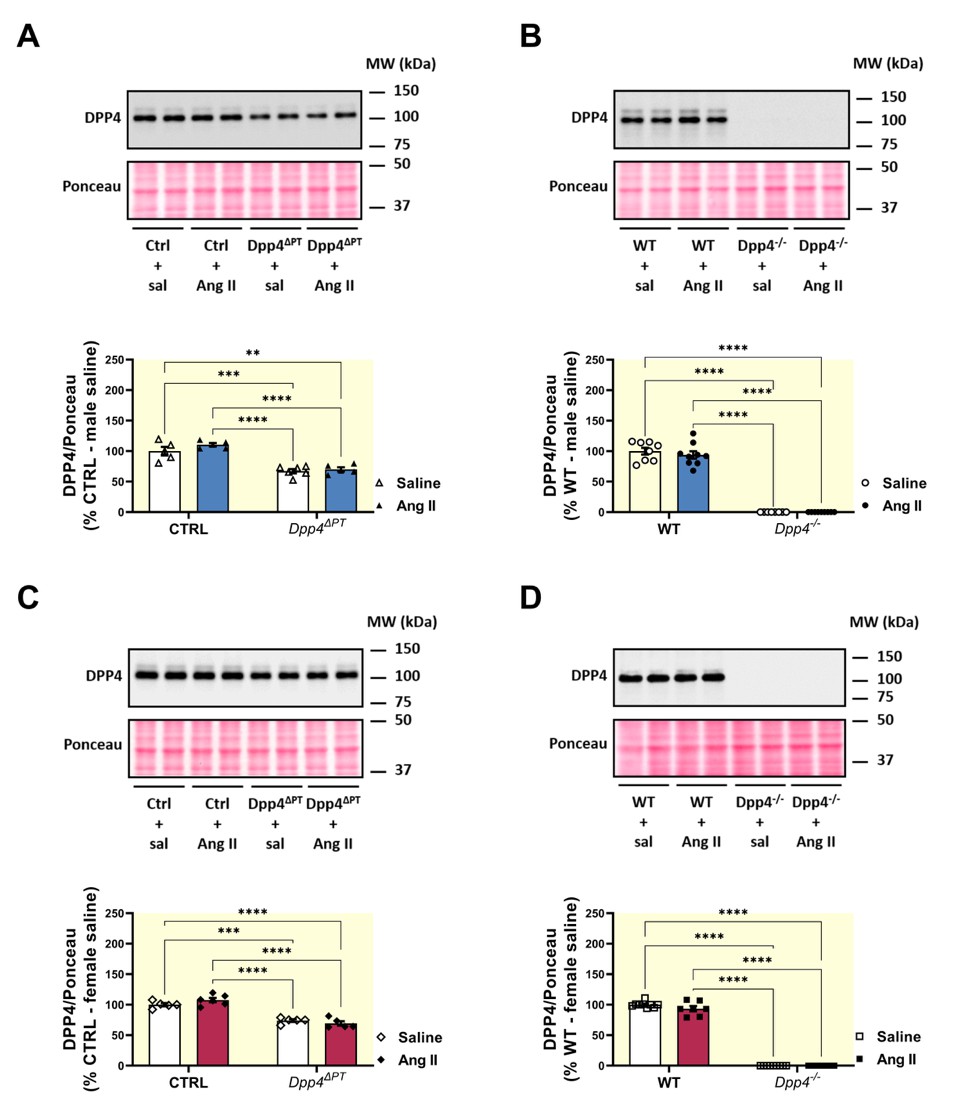


**Supplementary Figure S8 – Effect of acute Ang II administration on renal DPP4 protein abundance in male and female *Dpp4*^ΔPT^ and *Dpp4*^-/-^ mice.** DPP4 abundance was analyzed by immunoblotting in kidney homogenates (10 μg) from **(A)** male *Dpp4*^ΔPT^, **(B)** male Dpp4*^-/-^*, **(C)** female *Dpp4*^ΔPT^, and **(D)** female *Dpp4*^-/-^ mice. The densitometry results were normalized to Ponceau staining (~42 kDa) and expressed as % of CTRL or WT male/female. Data expressed as mean ± SEM. Data were tested for normality using the Shapiro-Wilk test. The experimental n ranged from 5 to 9. Statistical analysis was performed using two-way ANOVA followed by Tukey's post-test. **P < 0.01, ***P < 0.001 and ****P < 0.0001.


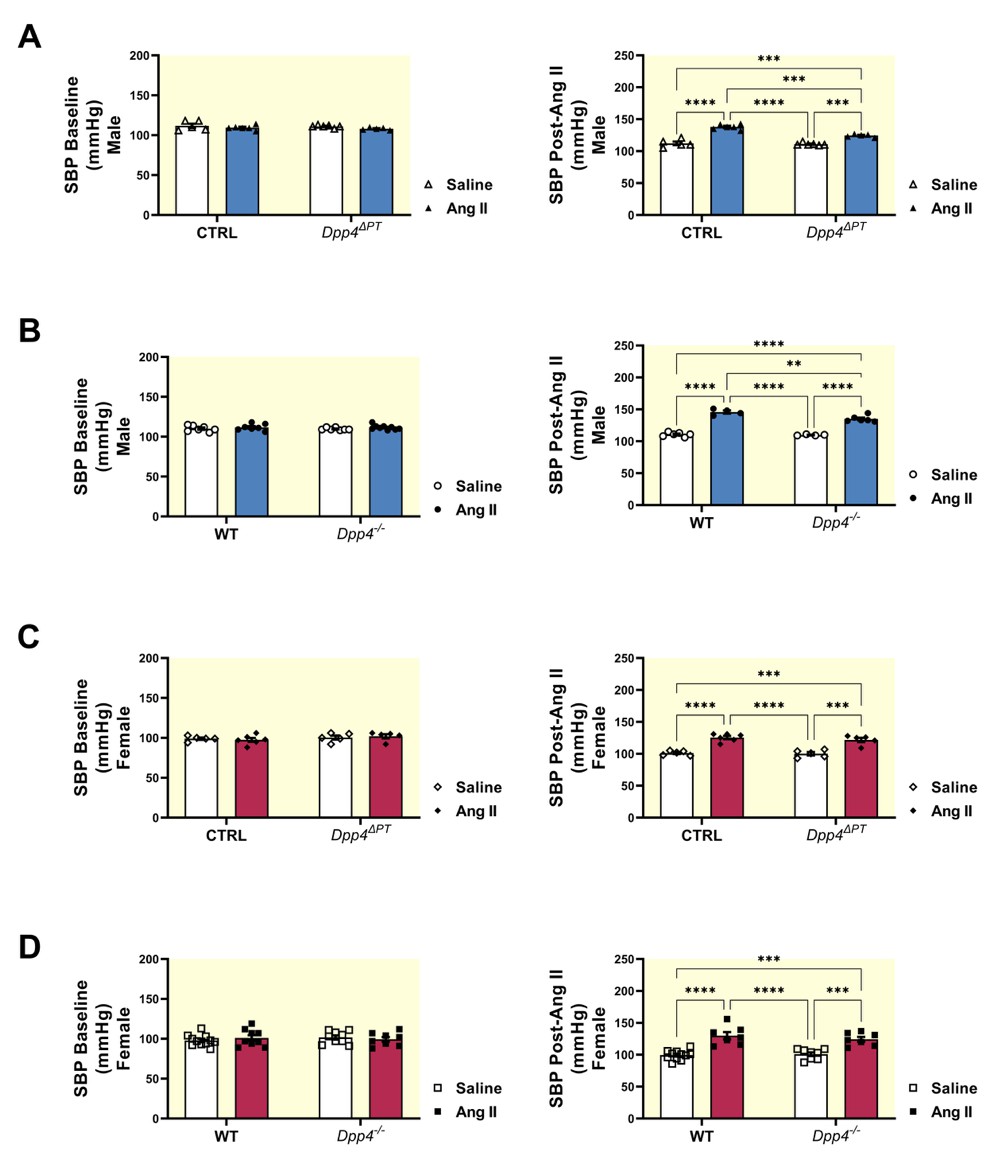


**Supplementary Figure S9 – Baseline and post-Ang II systolic blood pressure (SBP) in male and female *Dpp4*^ΔPT^ and *Dpp4*^-/-^ mice.** SBP was measured by tail-cuff plethysmography in *Dpp4*^ΔPT^, *Dpp4*^-/-^, and their respective male and female littermate controls. Panels **A-D** show baseline SBP (left) and SBP after 1 h of Ang II stimulation (right).  Data were tested for normality using the Shapiro-Wilk test. The experimental n ranged from 5 to 11. Statistical analysis was performed using two-way ANOVA followed by Tukey's post-test. **P < 0.01, ***P < 0.001 and ****P < 0.0001.


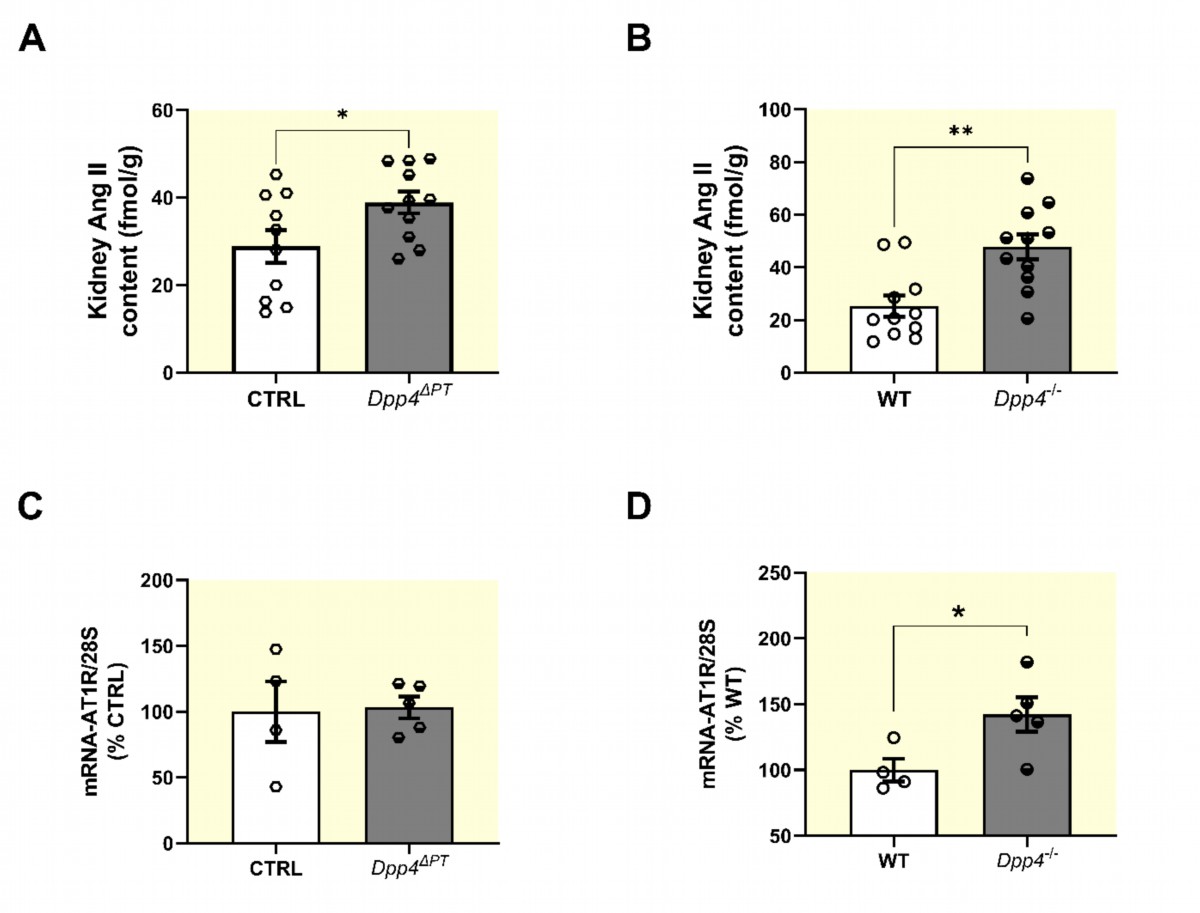


**Supplementary Figure S10 – Renal angiotensin II (Ang II) content and relative mRNA expression of AT1R in Dpp4^ΔPT^ and Dpp4^-/-^mice.** Renal Ang II levels were measured by enzyme-linked immunosorbent assay in **(A)** male CTRL and Dpp4^ΔPT^mice and **(B)**male WT and Dpp4^-/-^ mice. AT1R gene expression was assessed by reverse transcription quantitative PCR, normalized to 28S rRNA, and expressed as a percentage relative to the corresponding control group. Relative mRNA expression is shown for male **(C)** CTRL and Dpp4^ΔPT^ mice and **(D)** male WT and Dpp4^-/-^ mice. Data are expressed as mean ± SEM, with dots representing individual measurements. Data are expressed as mean ± SEM, with dots representing the % of CTRL or WT for each animal. Data were tested for normality using the Shapiro-Wilk test. The experimental n ranged from 4 to 5. Statistical analysis was performed using Student's T-test. *P < 0.05, **P < 0.01.

**FULL UNEDITED GELS**


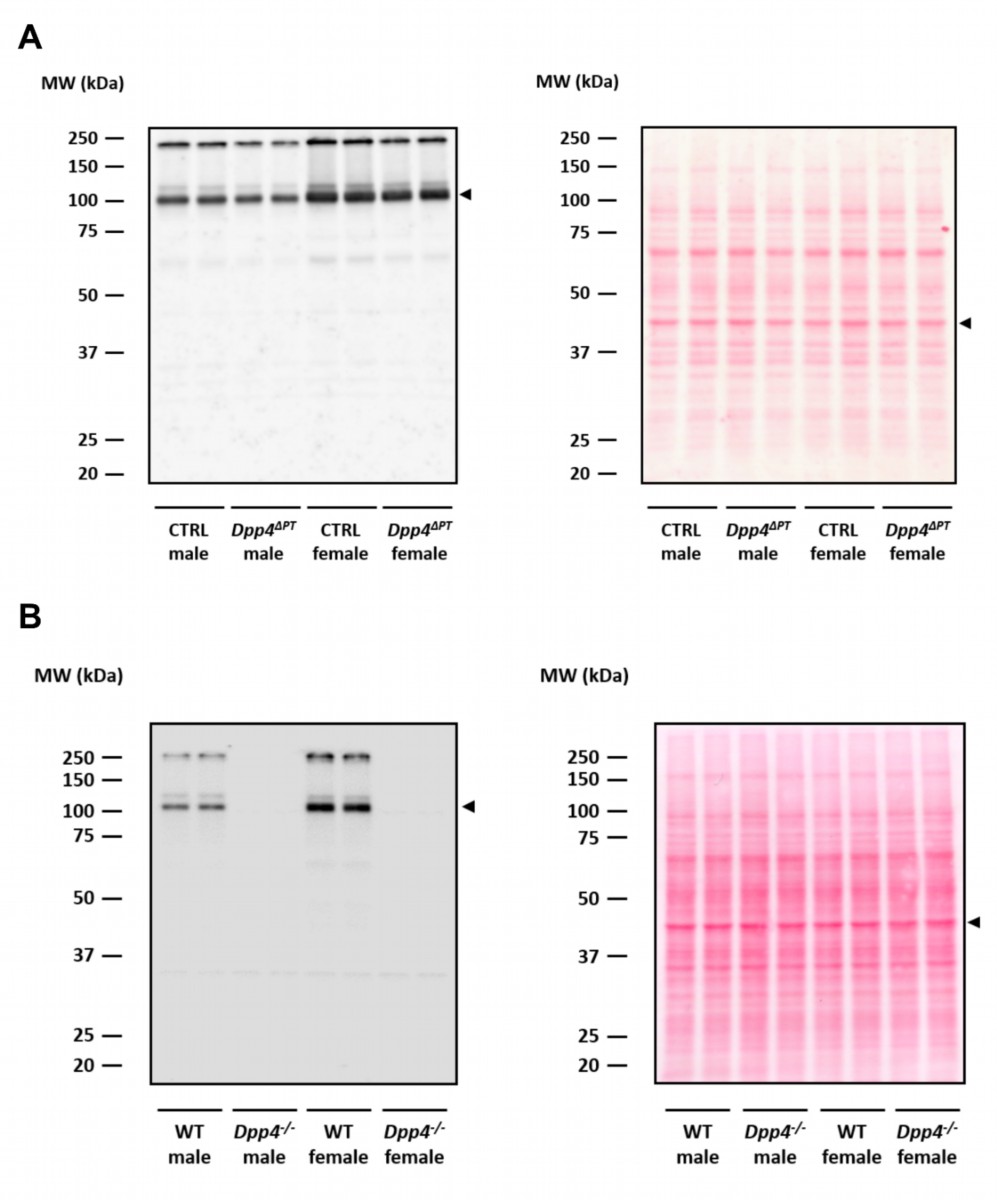


**Supplementary Figure S11.** Full unedited gel for Figure 1. **(A)** Left: Anti-DPP4 blot; Right: Ponceau staining (Figure 1A). **(B)** Left: Anti-DPP4 blot; Right: Ponceau staining (Figure 1B).


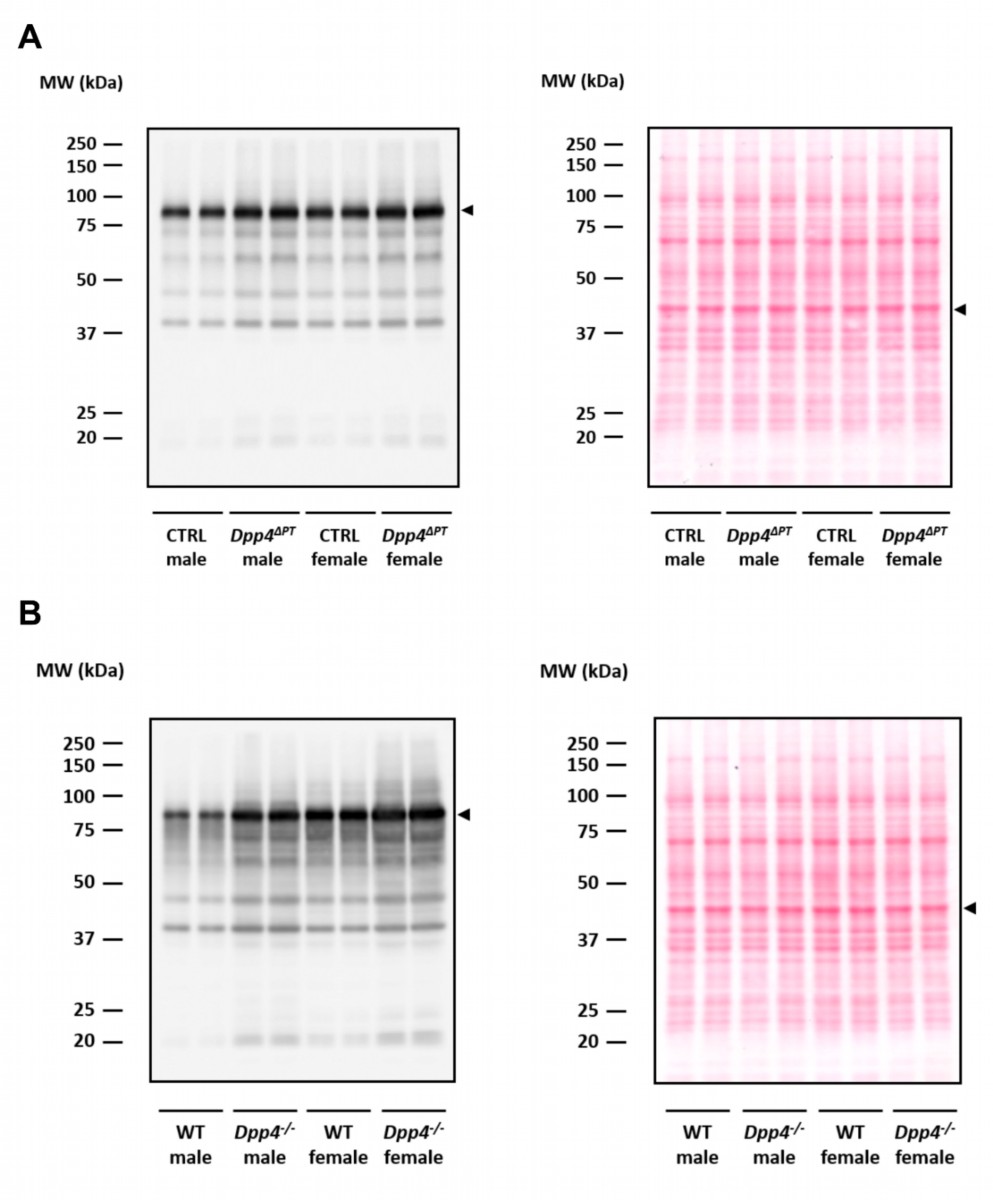

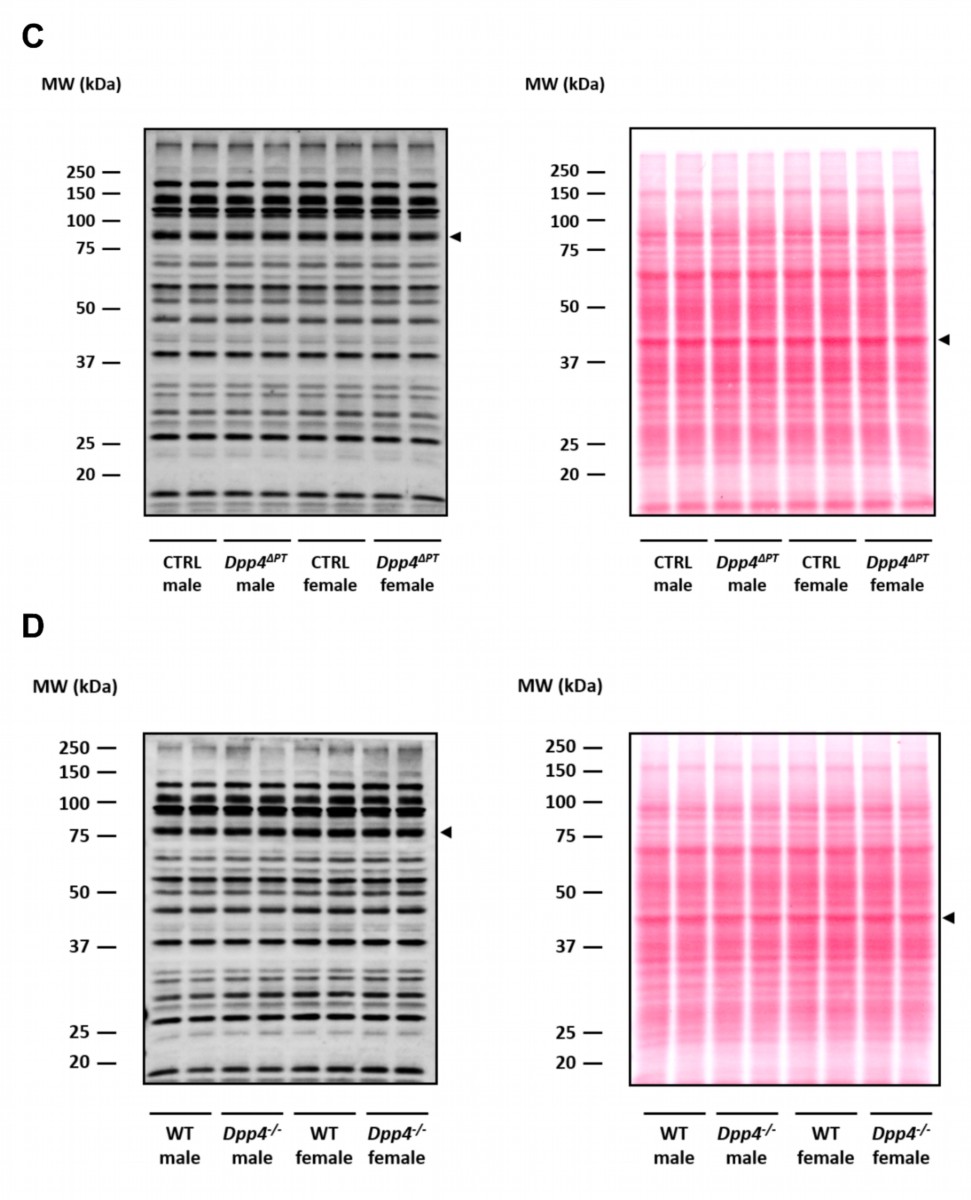


**Supplementary Figure S12.** Full unedited gel for Figure 3. **(A)** Left: Anti-pS552-NHE3 blot; Right: Ponceau staining (Figure 3A). **(B)** Left: Anti-pS552-NHE3 blot; Right: Ponceau staining (Figure 3D). **(C)** Left: NHE3 blot; Right: Ponceau staining (Figure 3A). **(D)** Left: NHE3 blot; Right: Ponceau staining (Figure 3D).


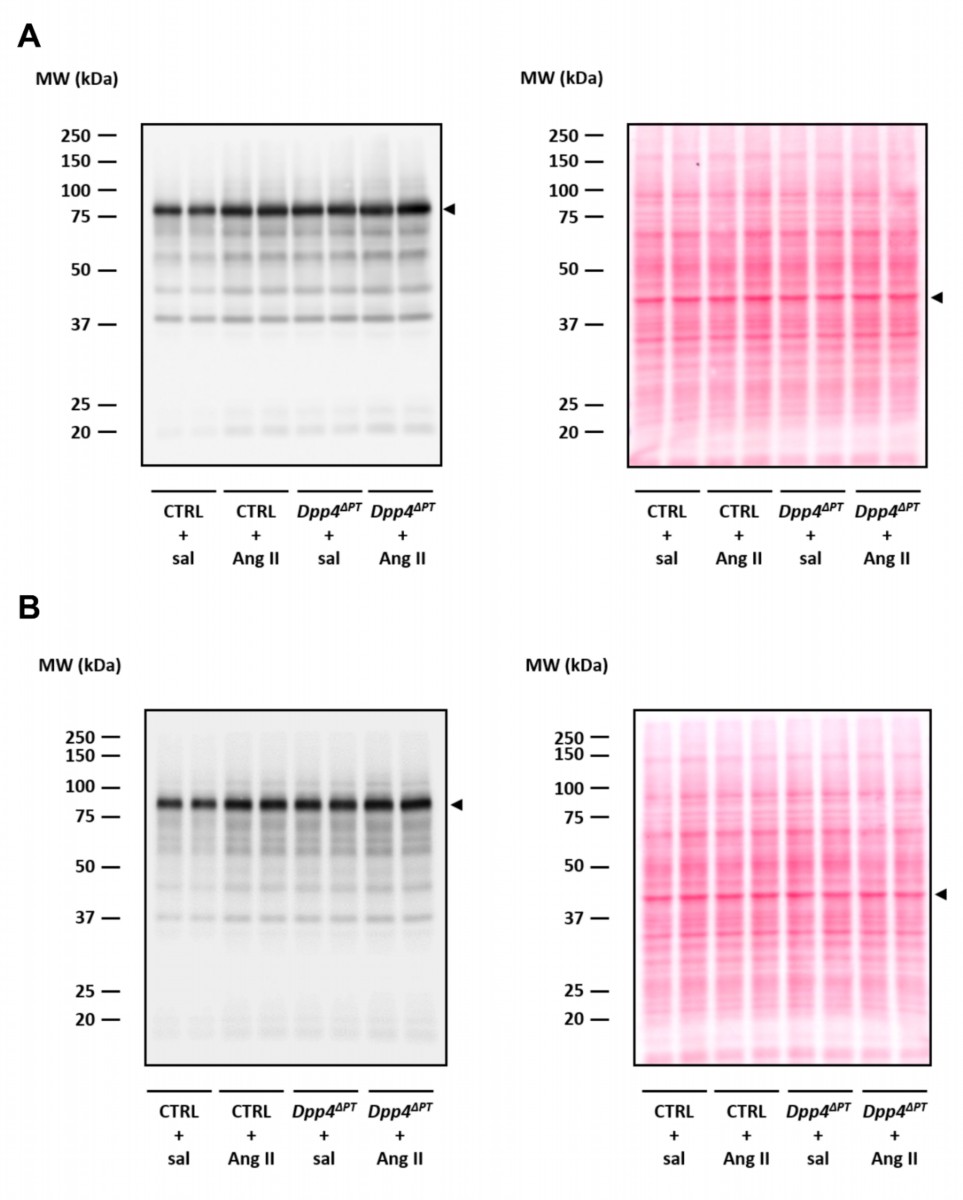

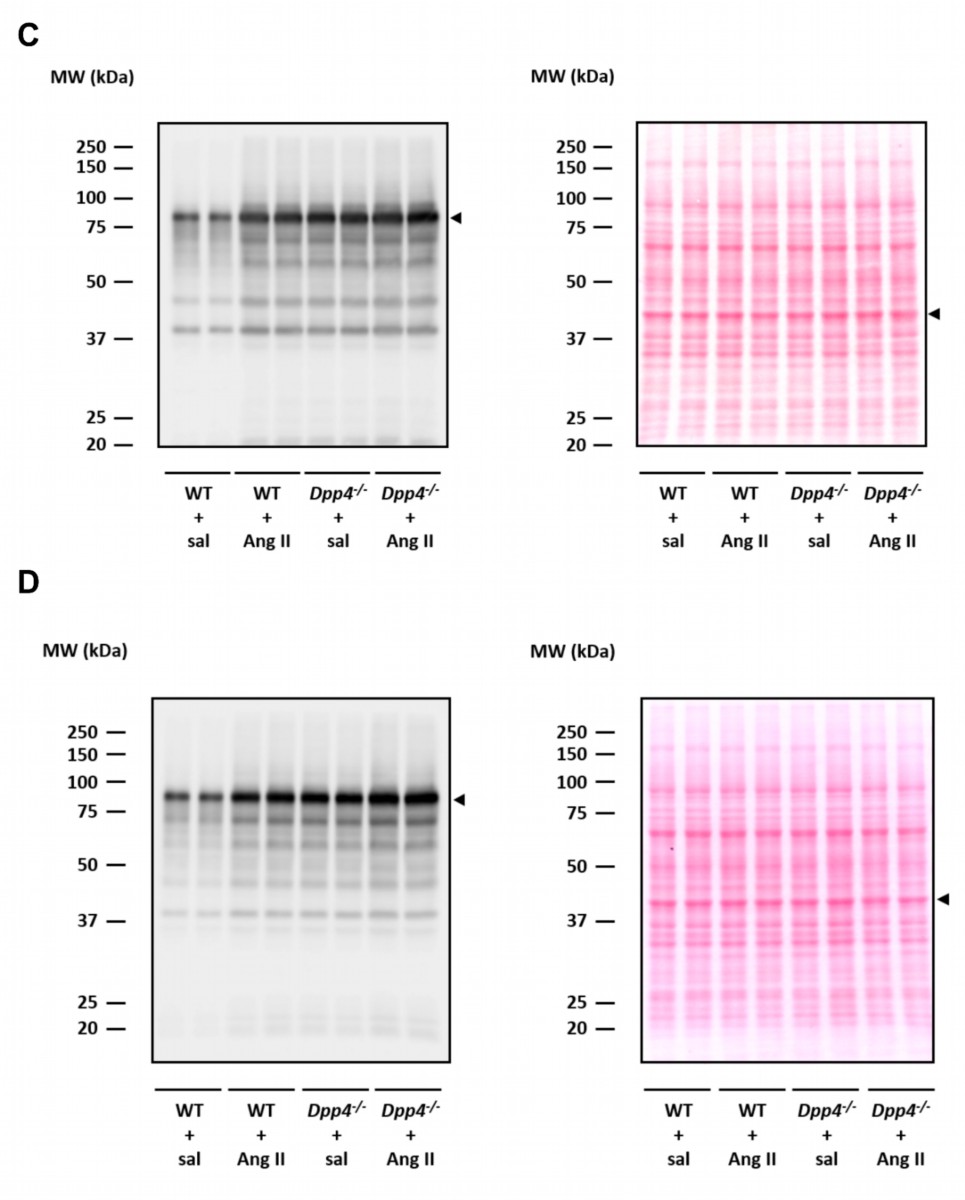

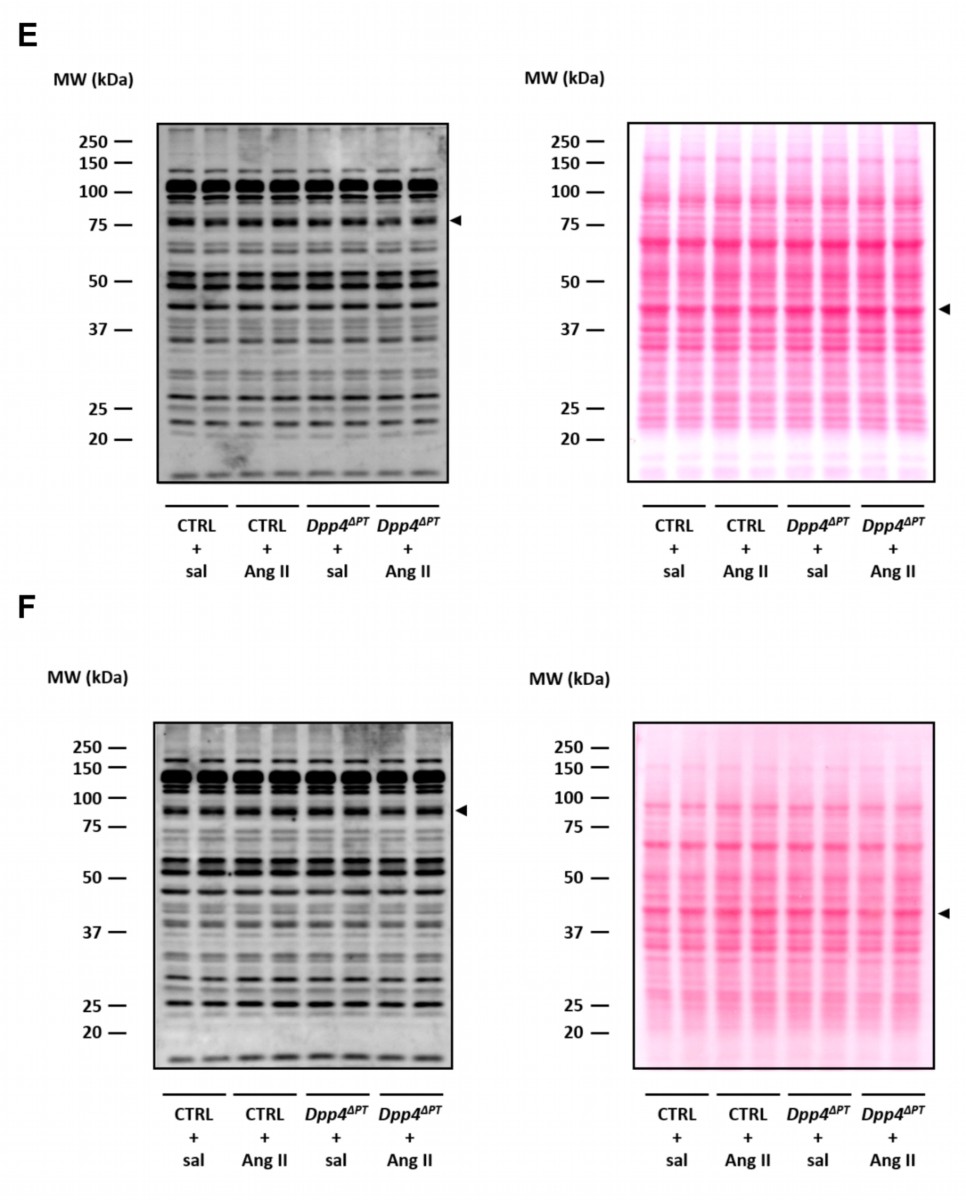

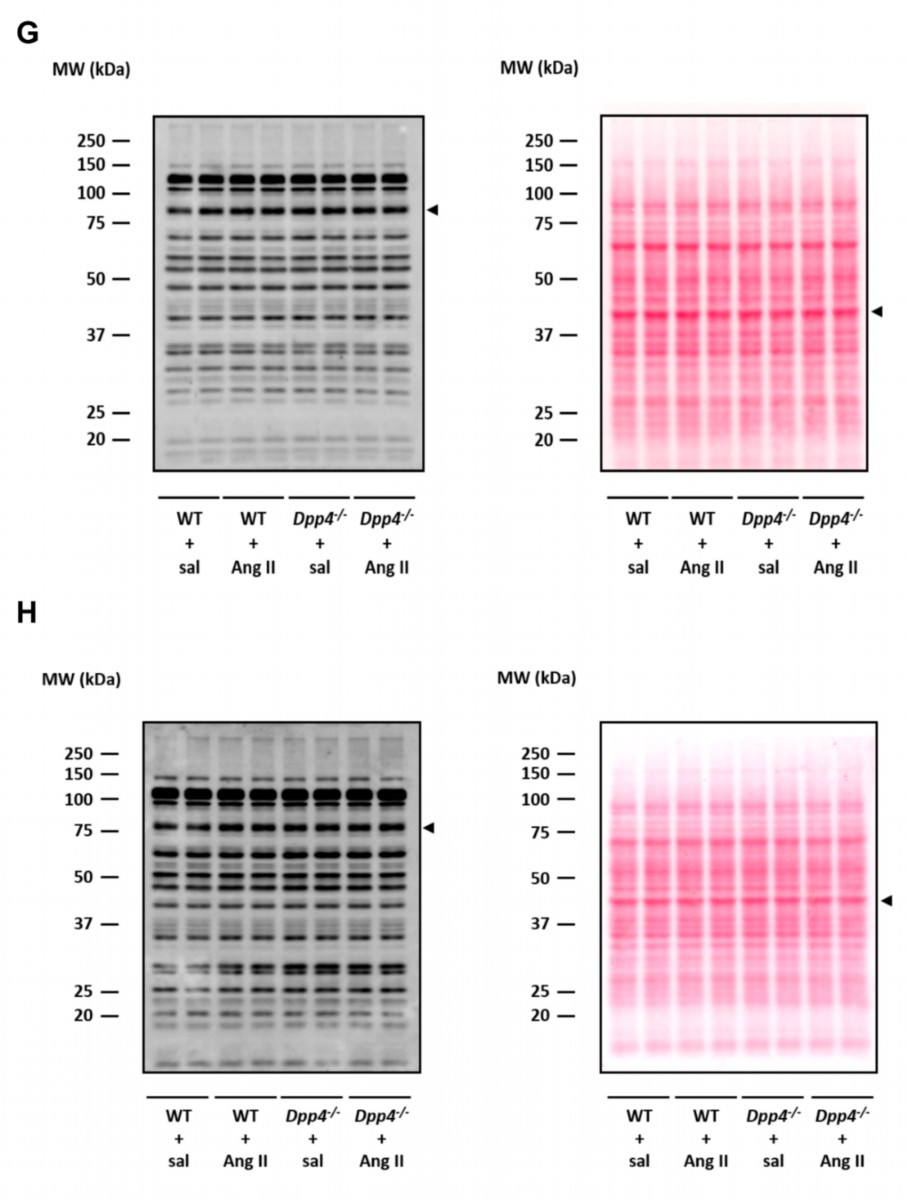


**Supplementary Figure S13.** Full unedited gel for Figure 6. **(A)** Left: Anti-pS552-NHE3 blot; Right: Ponceau staining (Figure 6A). **(B)** Left: Anti-pS552-NHE3 blot; Right: Ponceau staining (Figure 6D). **(C)** Left: Anti-pS552-NHE3 blot; Right: Ponceau staining (Figure 6G). **(D)** Left: Anti-pS552-NHE3 blot; Right: Ponceau staining (Figure 6J). **(E)** Left: NHE3 blot; Right: Ponceau staining (Figure 6A). **(F)** Left: NHE3 blot; Right: Ponceau staining (Figure 6D). **(G)** Left: NHE3 blot; Right: Ponceau.(Figure 6G). **(H)** Left: NHE3 blot; Right: Ponceau staining (Figure 6J).


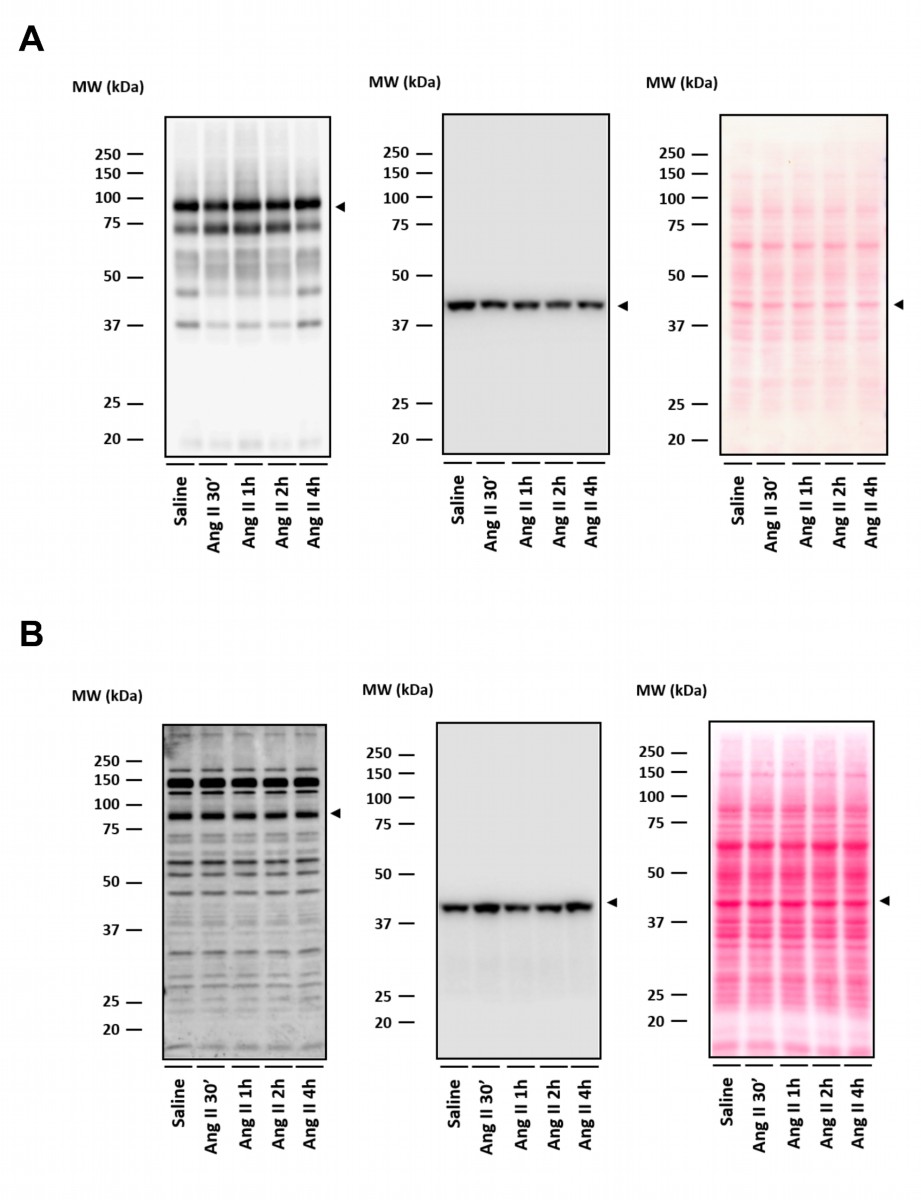


**Supplementary Figure S14.** Full unedited gel for Supplementary Figure S4. **(A)** Left: Anti-pS552-NHE3 blot; Middle: β-actin blot; Right: Ponceau staining for male WT kidney homogenates (Supplementary Figure S4B). **(B)** Left: NHE3 blot; Middle: β-actin blot; Right: Ponceau staining (Supplementary Figure S4C).


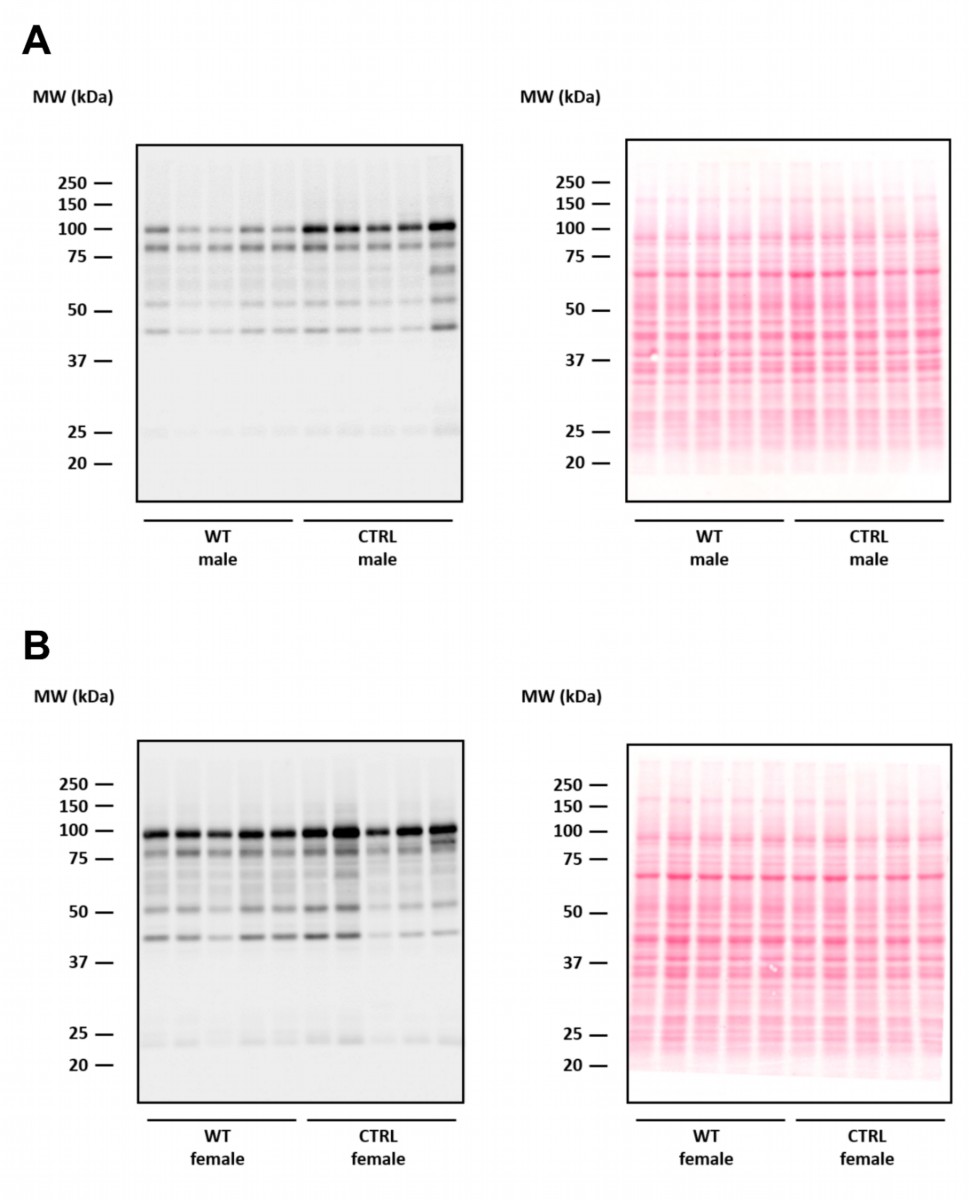


**Supplementary Figure S15 -** Full unedited gel for Supplementary Figure S5. **A)** Left panel: pS552-NHE3 and Right panel: ponceau staining, in CTRL and WT male mice kidneys homogenates (Supplementary figure S5A). **B)** Left panel: pS552-NHE3 and Right panel: ponceau staining, in CTRL and WT female mice kidneys homogenates (Supplementary figure S5B).


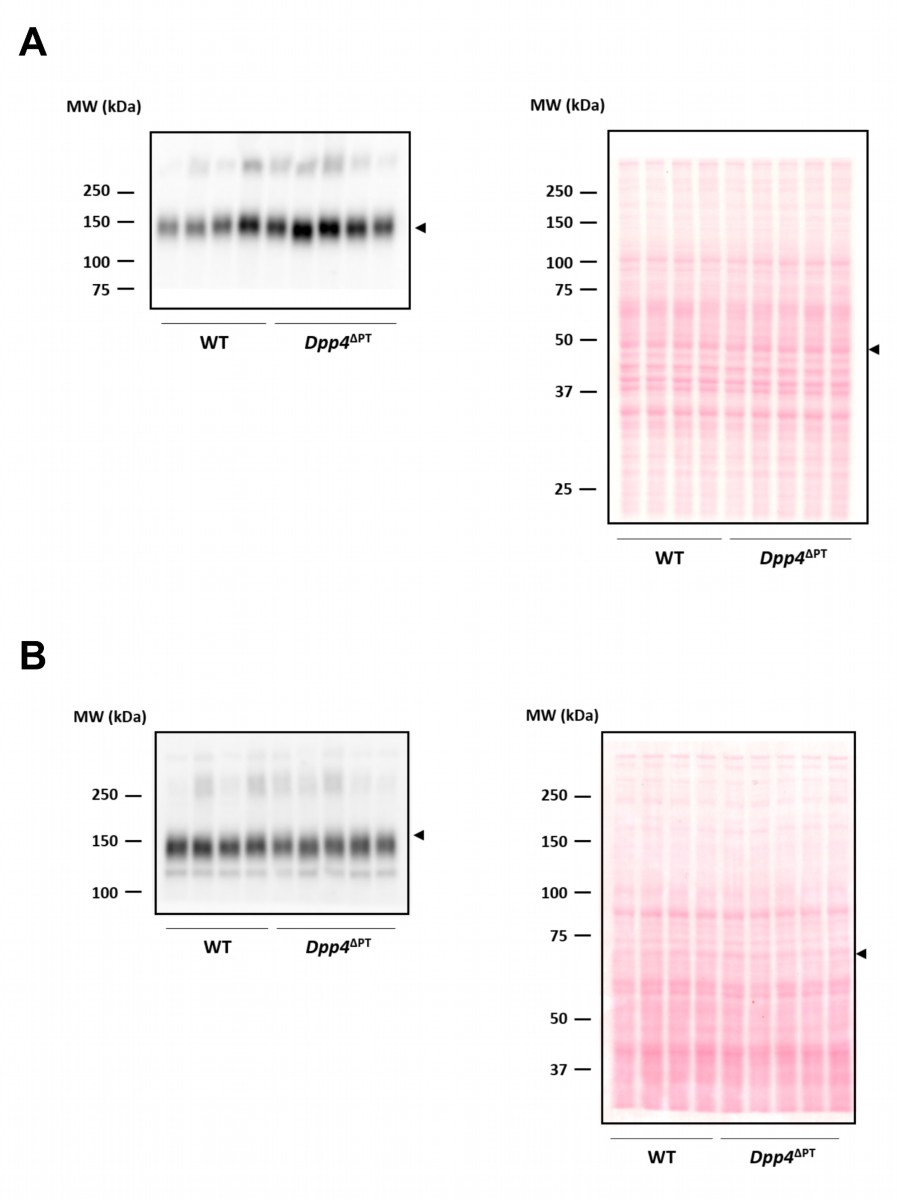

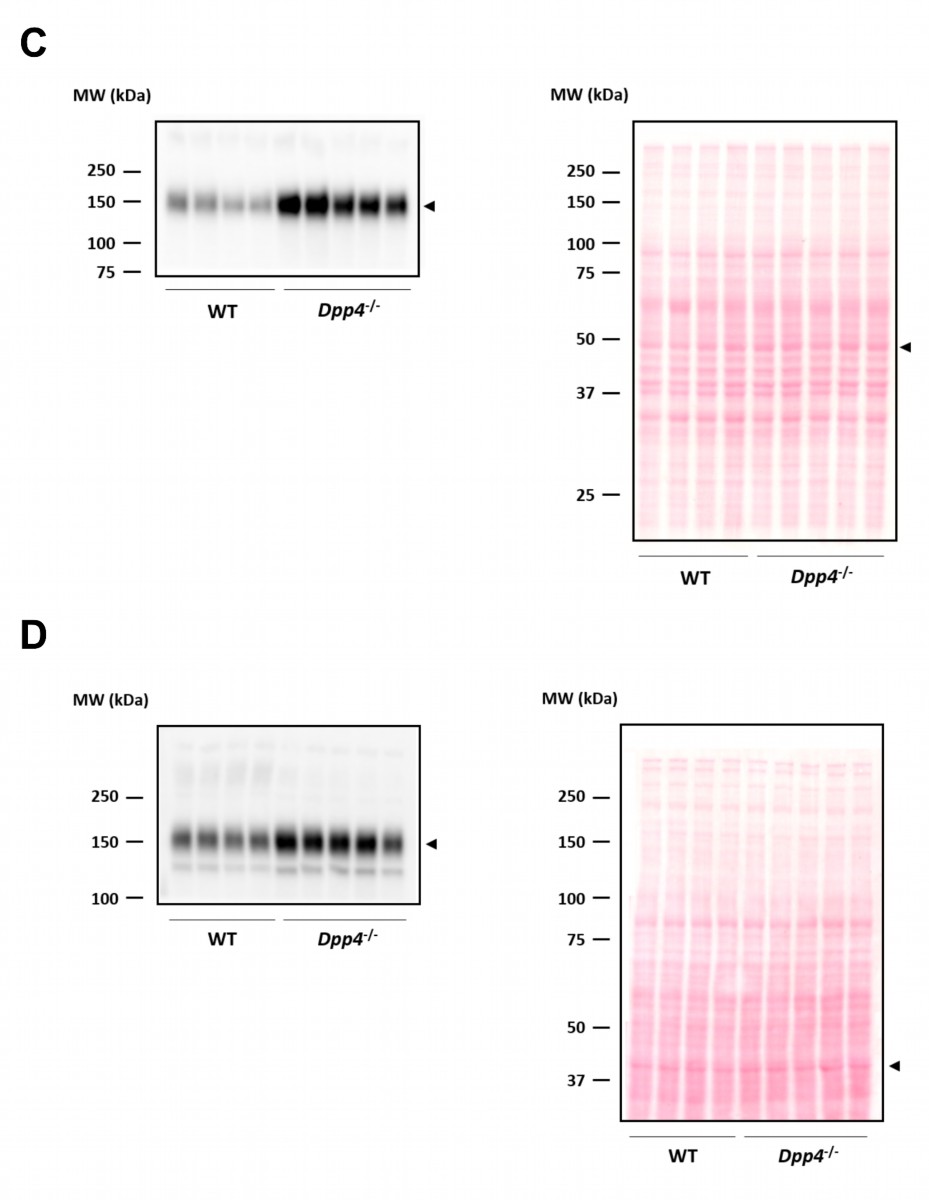


**Supplementary Figure S16.** Full unedited gel for Supplementary Figure S6. **(A)** Left: pT53-NCC blot; Right: Ponceau staining *Dpp4^ΔPT^* mice (Supplementary Figure S6A). **(B)** Left: pT53-NCC blot; Right: Ponceau staining *Dpp4^-/-^* mice (Supplementary Figure S6B). **(C)** Left: total NCC blot; Right: Ponceau staining *Dpp4^ΔPT^* mice (Supplementary Figure S6C). **(D)** Left: total NCC blot; Right: Ponceau staining *Dpp4^-/-^* mice (Supplementary Figure S6D).


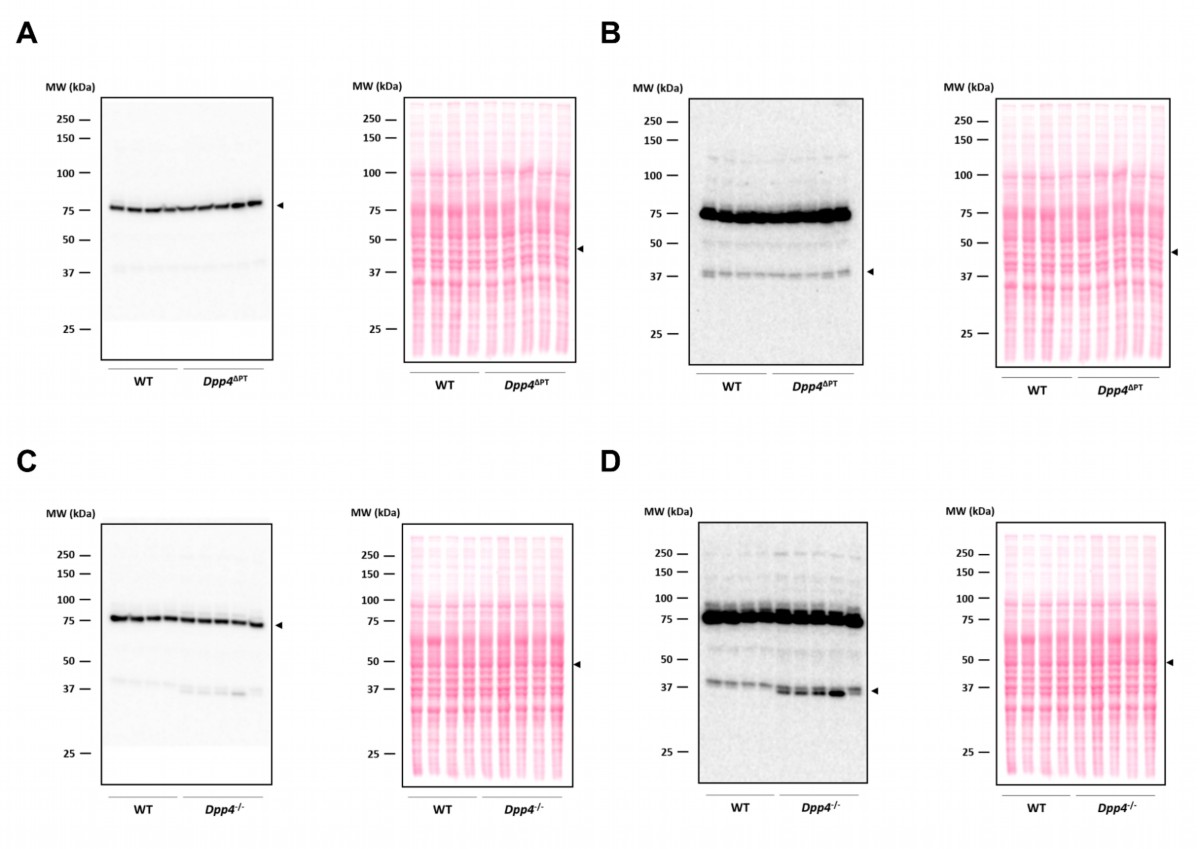

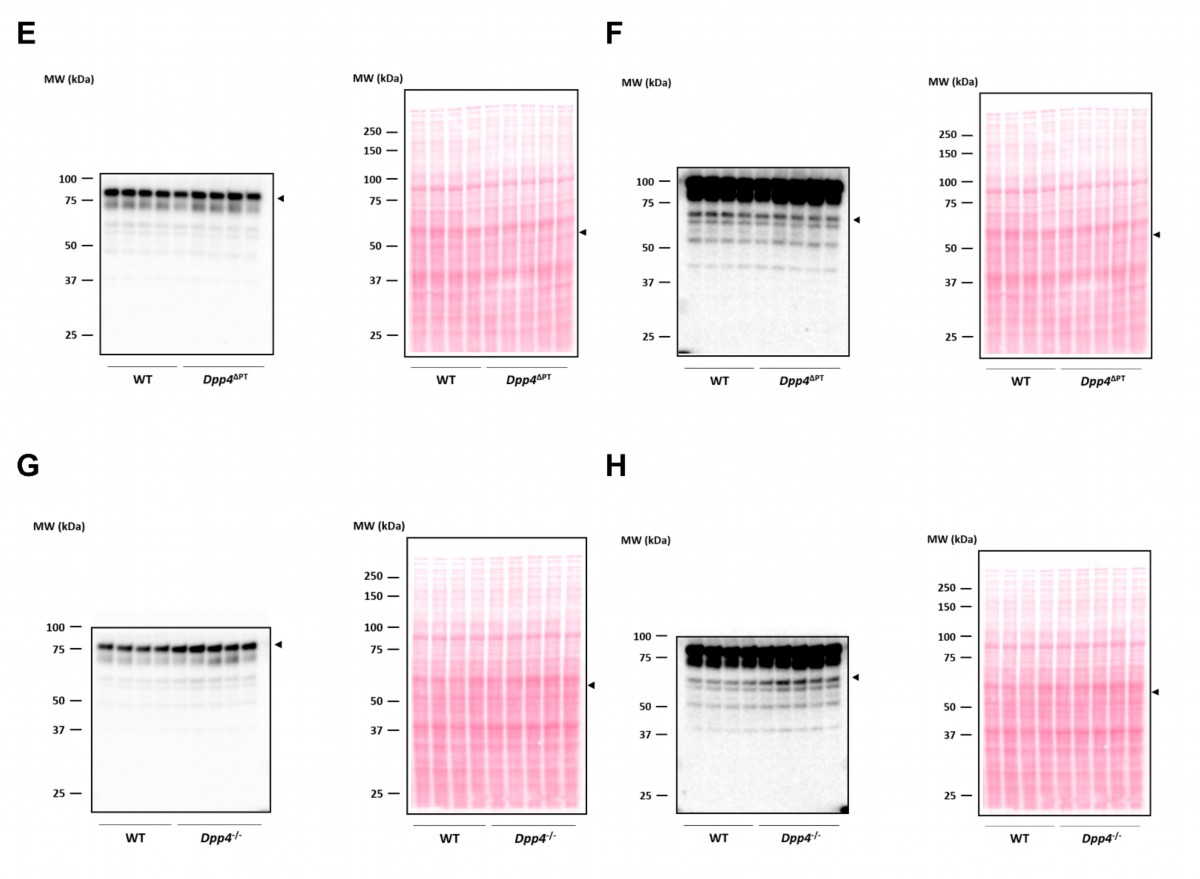


**Supplementary Figure S17.** Full unedited gel for Supplementary Figure S7. **(A)** Top left: full-length α ENaC blot *Dpp4^ΔPT^* mice (Supplementary Figure S7A); **(B)** Top right: cleaved α ENaC blot *Dpp4^ΔPT^* mice (Supplementary Figure S7B); **(C)** Bottom left: full-length α ENaC blot *Dpp4^-/-^* mice (Supplementary Figure S7C); **(D)** Bottom right: cleaved α ENaC blot *Dpp4^-/-^* mice (Supplementary Figure S7D); **(E)** Top left: full-length γ ENaC blot *Dpp4^ΔPT^* mice (Supplementary Figure S7E); **(B)** Top right: cleaved γ ENaC blot *Dpp4^ΔPT^* mice (Supplementary Figure S7F); **(C)** Bottom left: full-length γ ENaC blot *Dpp4^-/-^* mice (Supplementary Figure S7G); **(D)** Bottom right: cleaved γ ENaC blot *Dpp4^-/-^* mice (Supplementary Figure S7H).

**
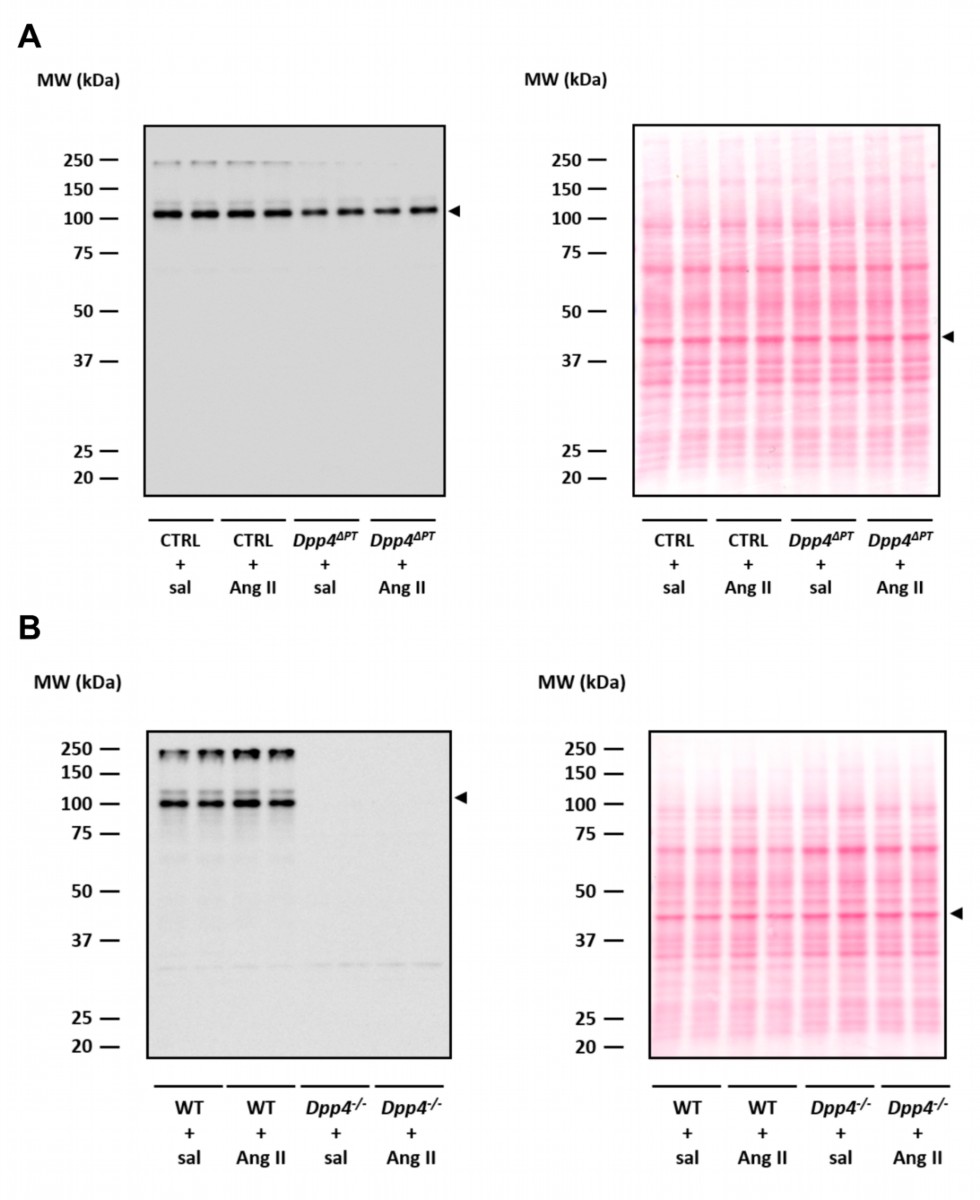
**
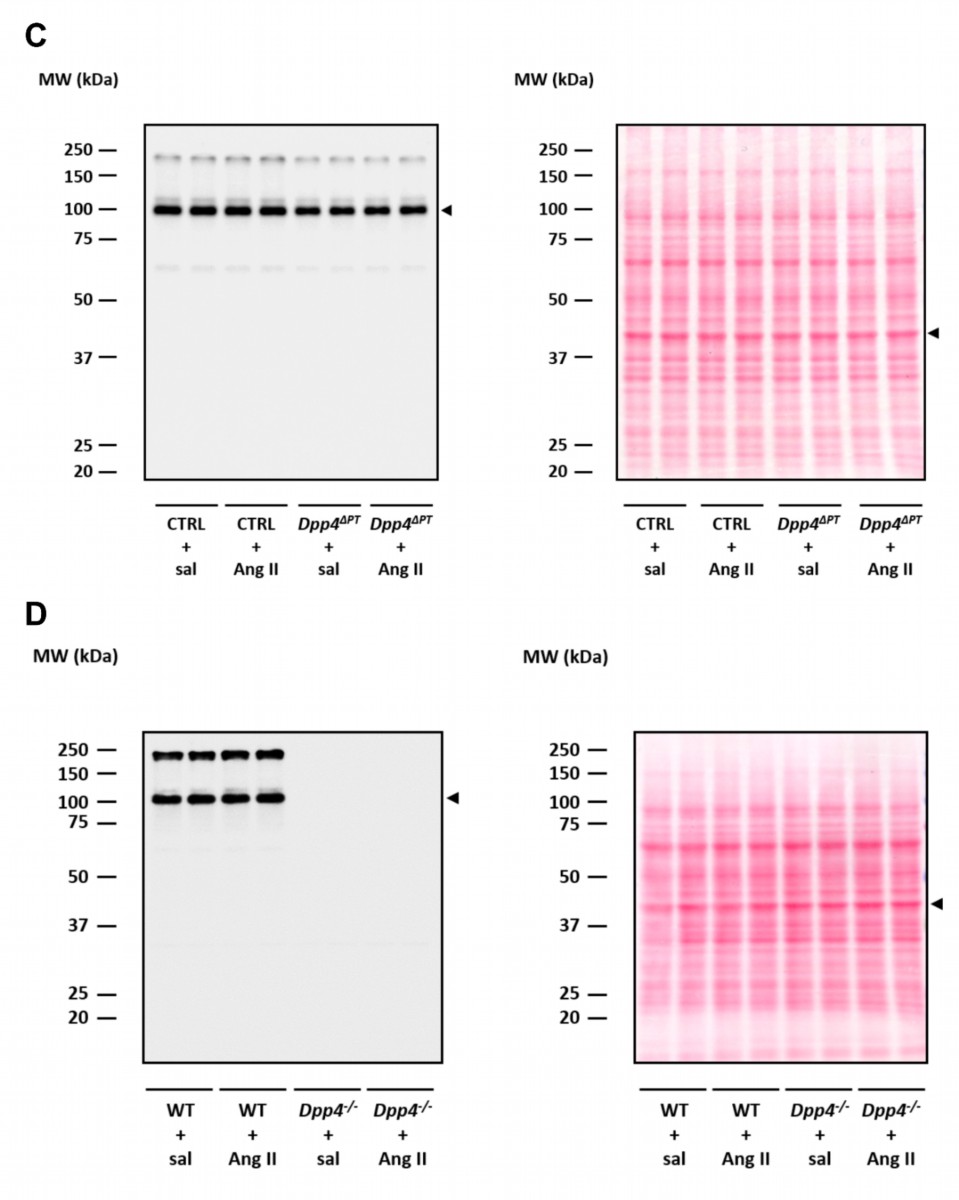


**Supplementary Figure S18.** Full unedited gel for Supplementary Figure S8. **(A)** Left: DPP4 blot; Right: Ponceau staining (Supplementary Figure S8A). **(B)** Left: DPP4 blot; Right: Ponceau staining (Supplementary Figure S8B). **(C)** Left: DPP4 blot; Right: Ponceau staining (Supplementary Figure S8C). **(D)** Left: DPP4 blot; Right: Ponceau staining (Supplementary Figure S8D).
